# Emissions and Cost Trade-Offs of Time-Matched Clean Electricity Procurement under Interannual Weather Variability: A Case Study of Hydrogen Production

**DOI:** 10.1021/acs.est.6c00988

**Published:** 2026-07-06

**Authors:** Michael A. Giovanniello, Dharik S. Mallapragada

**Affiliations:** † MIT Energy Initiative, 2167 Massachusetts Institute of Technology, Cambridge, Massachusetts 02139, United States; ‡ Chemical and Biomolecular Engineering Department, Tandon School of Engineering, 34242 New York University, Brooklyn, New York 11201, United States

**Keywords:** emissions accounting, consequential emissions, capacity expansion modeling, hourly time-matching, demand flexibility

## Abstract

Regulators and voluntary corporate sustainability efforts are increasingly adopting time-matching requirements (TMRs) for clean electricity procurement for large loads, such as data centers, and electricity-intensive fuel production, such as hydrogen. We use a stochastic capacity expansion model (CEM) framework to assess how interannual weather variability affects the cost and emissions impact of procurement-driven infrastructure to meet annual and hourly TMRs using the case study of a grid-connected hydrogen producer in Texas. Our approach, which relies on co-optimizing investments and hourly operations over nine weather scenarios, reveals that hourly TMR comes at a higher cost premium compared to annual TMR than previously estimated by single-scenario deterministic modeling, while emissions outcomes remain directionally consistent. Demand flexibility and partial hourly TMR (80–90%) lowers the cost premium while preserving emissions benefits. We further examine how binding renewable portfolio standards (RPS) interact with TMR costs and emissions outcomes. When an RPS is applied to non-H_2_ electricity demand, annual TMR reduces emissions comparably to hourly TMR at a lower cost. Incorporating H_2_-related electricity demand directly into the RPS constraint, rather than imposing a separate TMR, achieves similar emissions outcomes at still lower cost, suggesting that TMR-based clean electricity procurement—particularly hourly matching—offers limited additional value in regions with stringent grid decarbonization policies.

## Graphical abstract

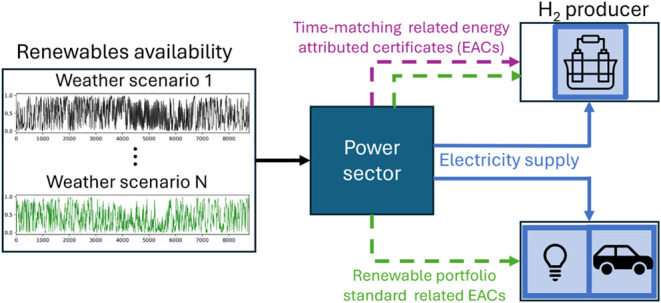

## 1 Introduction

Electricity consumers—both large and small—are showing increasing interest in managing their electricity-related carbon footprint (also known as Scope 2 emissions) through additional procurement of clean electricity. By the end of 2024, voluntary procurement by consumers in the U.S. amounted to over 70 GW of variable renewable energy (VRE) generation capacity^1^—for context, total installed solar and wind capacity by the end of 2023 was 95 and 149 GW, respectively.^2^ As electricity demand for data centers to support artificial intelligence (AI) applications is expected to grow rapidly,^3^ this growth could also accelerate corporate clean energy procurement as firms look to meet their sustainability goals.

In addition to voluntary procurements, regulatory requirements are also driving electricity consumers to procure clean electricity. For example, electricity-based hydrogen (H_2_) producers in the U.S. and E.U. must meet specific eligibility criteria to receive emissions-indexed production tax credits.^4,5^ These include matching their electricity consumption with an equivalent amount of clean electricity from newly added, or so-called “additional,” generation capacity, initially on an annual basis, and eventually on an hourly basis. Variants of these so-called time-matching requirements (TMRs) are also being considered in ongoing revisions of electricity-related emissions accounting protocols that many private sector firms adopt to manage and report their electricity-related emissions footprint.^6^

Previous assessments of alternative clean electricity procurement strategies have used two methodological approaches: (a) *Demand-centric modeling*,^7–11^ which evaluates the necessary co-located clean electricity investments to meet consumer demand, and potentially offset grid electricity consumption or its associated emissions, while treating the consumer as a price-taker; and (b) *Grid-centric modeling*,^5,12–16^ which examines the power system impacts of consumer-driven clean electricity procurement by co-optimizing grid-wide investments and operations to meet both aggregate demand and consumer clean electricity targets under various scenarios. Both modeling approaches have been extensively used to study the economics and emissions impacts of electricity-based H_2_ production.^17–21^ The discussion below, however, focuses on studies that consider clean electricity procurement strategies, of which H_2_ production is a common but not exclusive application. We also exclude discussion of systems involving colocated VRE generation and electrolytic H_2_ production without grid connectivity, as the alternative clean electricity procurement strategies considered here are not relevant for those configurations.

To date, demand-centric modeling studies have focused on the costs of more stringent hourly time-matching vs. less stringent annual time-matching, as well as the impact of technological and spatial diversification.^7–9^ For example, Casa Ferrus et al.^9^ compare the levelized cost of hydrogen (LCOH) production under two procurement strategies sourcing VRE supply from a single asset or from a spatially diversified portfolio of assets—and find cost savings with the portfolio approach. While many such studies rely on a single weather year to model VRE generation and system operations, a few demand-centric analyses have also accounted for VRE resources’ interannual variability.^7,8^ Such interannual variation has been shown to substantially influence investment needs, particularly for energy storage, in studies focused on power system decarbonization using 100% VRE supply.^22–24^ Interannual VRE variation is particularly important for clean electricity procurement since consumers often are looking to sign multiyear contracts with VRE generators.

Many grid-centric modeling studies have used capacity expansion models (CEMs) to quantify the power system-wide impacts and consumer-level costs and emissions under alternative clean electricity procurement strategies. Unlike demand-centric models, grid-centric analyses using CEMs capture competition among consumers for limited clean electricity resources and evaluate how this competition affects clean electricity procurement outcomes, as well as system-level cost and emissions outcomes. These models also account for multiple revenue streams available to grid-connected VRE resources, including those arising from contracts with individual consumers and from sales of energy and capacity to the grid. By comparing system-level counterfactuals with and without procurement strategies and quantifying the revenue streams earned by procured resources, these models provide a more realistic measure of *additionality*, which is the causal relationship between consumer procurement and clean electricity deployment. Additionality measures whether clean electricity procurement actions actually cause new clean electricity generation to be added to the power system, beyond what would have been built anyway. For instance, Giovanniello et al.^12^ used a CEM-based framework to highlight how alternate definitions of additionality can change the emissions outcomes associated with electricity-based H_2_ production that employs annual time-matching. The study found that a weaker additionality definition, which classifies any new generation as additional, can result in higher emissions. In contrast, more stringent definitions that require the new generation to not have been built without the consumer-driven TMR constraint, were found to result in emissions outcomes comparable to those achieved with hourly TMR.^12^ Here, it is important to note that definitions of additionality vary across studies, depending on whether they are tracking emissions, generation, or revenue impacts of clean electricity procurement and how they are computed. See Giovanniello et al.^12^ for a discussion on the latter aspect of defining additionality.

Grid-centric modeling studies have identified several insights into TMR as a strategy for clean electricity procurement: (a) hourly matching is likely to lead to lower system-wide emissions than annual matching but is more expensive;^12–14,25^ (b) grid-level policies, such as binding requirements on shares of low-carbon or VRE generation, lower the emissions impact of the less granular annual matching without significantly impacting its cost;^12,16,25^ (c) the availability of clean firm resources and long-duration energy storage reduces the cost premium of more granular hourly matching,^13,25,26^ potentially paving the way for their scale-up through early adoption by willing consumers using such procurement strategies;^27^ and (d) the region-specific impacts of spatial and temporal matching requirements can vary significantly due to prevailing grid conditions and constraints on short-term VRE expansion.^5,16^

The studies cited above use models that consider only a single weather scenario for annual power system operations and do not account for interannual variations in VRE availability. In practice, however, consumers are interested in clean electricity procurement over longer time frames (e.g., 10 or 20 years), for which interannual variation in weather and its impact on VRE availability becomes relevant. Sizing clean electricity procurements based on a single weather scenario could lead to excess generation relative to demand in years of higher VRE availability and deficits in years with lower-than-anticipated VRE availability. These studies may also undervalue storage resources and diversification of the VRE resource portfolio, which help to manage temporal mismatches between demand and the output of any single clean electricity resource. As noted earlier, while interannual weather variation has been examined in the context of islanded H_2_ production system design^7,8^ and broader grid decarbonization studies,^22–24^ its effect on the system-wide impacts of clean electricity procurement strategies has, to our knowledge, not been previously considered.

Here, we investigate the impact of interannual VRE variation on the investments needed to meet either annual or hourly TMR for a grid-connected electricity-based H_2_ producer to meet baseload H_2_ demand. We use a CEM to evaluate the cost-optimal grid and procurement-driven infrastructure investments in VRE capacity, battery energy storage, H_2_ storage, and electrolyzer capacity that are needed to meet demand and enforced TMRs (annual or hourly), as well as system operations. We consider both (a) a single weather scenario (“Deterministic”) and (b) nine alternative weather scenarios (“Stochastic”). We then assess the robustness of the optimized infrastructure mix by testing its performance against 10 weather scenarios that are not included in the investment planning phase. Our case study, based on the Texas grid, explores a range of technological and policy scenarios involving different levels of energy storage availability and varying degrees of compliance with the enforced TMR for electrolytic hydrogen demand. We also examine how grid-wide decarbonization policies, specifically renewable portfolio standards (RPS) applied to existing demand, affect system emissions outcomes and the composition of clean electricity procurement portfolios for meeting annual and hourly TMRs under interannual VRE variability. We also evaluate the impact of incorporating additional electricity demand from H_2_ production into an existing RPS policy rather than via separate TMR-based procurement. Finally, we undertake a revenue analysis of VRE resources deployed under scenarios with and without RPS to demonstrate how revenue metrics can be used to assess the additionality of resources in real-world settings.

## 2 Methods

### 2.1 Model Overview

Our analysis uses the open-source CEM, DOLPHYN,^28^ which is formulated as a linear program (LP) for this study, to minimize the investment and operation cost of electricity and H_2_ infrastructure while respecting constraints related to (a) system operation including meeting specified hourly H_2_ and electricity demands, (b) technology operation, and (c) policy requirements either at the technology or system level (see Table S1). DOLPHYN is capable of modeling transmission constraints; however, as a simplification, we ignore electricity and hydrogen transmission constraints in this study. This effectively assumes that demand and supply are balanced at the system level without accounting for any costs/constraints associated with the transmission of electricity and H_2_ between supply and demand. We provide a full description of the key model constraints (Table S1 ) along with the solution approach (S1.5) in the Supporting Information (SI), with the code available on Github^28,29^ .

Our analysis relies on two different CEM versions: (a) *a deterministic model*, where we co-optimize investments and operations while considering system operations based on a single weather scenario at an hourly resolution, and (b) *a stochastic model*, where we co-optimize investments and operations while considering system operations based on nine different weather scenarios at an hourly resolution. Throughout the analysis, ″weather scenario″ refers to the weather year that corresponds to the availability time series for wind and solar resources. Exogenous electricity and H_2_ demand are held constant across weather scenarios.

Equation 1 shows the objective function of the stochastic model, which is composed of two parts: (1) the annualized investment cost of new resources (*k* ∈ *K*, first term) and (2) the expected operating costs for each weather scenario (*s*∈S), which includes annual fixed (2nd term) and variable operating costs (3rd term) for both existing and new resources as well as costs for load shedding (4th term). In eq 1, *K* is the set of all new resources, *T* is the set of hours of the year, *G* is the set of all existing and new resources, and *NSE* is the cost associated with nonserved energy for demand type *D*. Note that the model objective function covers both the electricity and hydrogen sectors; however, for simplicity, we have written the objective function in a sector-agnostic manner. For the stochastic model, the annual operating costs of each weather scenario are weighted equally in the model objective function, i.e., σ_*s*_ = 1/*N*_scen_, where *N* **_*scen*_** is the number of weather scenarios considered. For the deterministic model, σ_*s*_ = 1.

objective=Σk∈Kcapk*inv_cost⁡k+Σs∈sσsΣt∈t(Σg∈G(fixed_cost⁡g(s,t)+var⁡_cost⁡g(s,t)+NSd∈DEd(s,t)) 1

Equations 2–3 describe the constraints enforcing hourly and annual time-matching of electricity consumption for H_2_ production. For each hour, eq 2 requires that the electrolyzer power consumption, equal to tonnes of H_2_ produced in that hour times the power required per tonne (λ^Ely^), multiplied by the degree of hourly compliance (α_TMR_) must be less than or equal to generation from contracted VRE generation (i.e., PPA resources, *g* ∈ TMR_*g*_) plus net injection from eligible battery storage (*b* ∈ TMR*_b_*). Here, α_TMR_ reflects the fraction of H_2_-related electricity consumption that needs to be matched by supply from PPA resources.

2 $$\sum_{g\in {\mathrm{TMR}}_{g}}{\mathrm{gen}}_{g,t}+\sum_{s\in {\mathrm{TMR}}_{b}}{\mathrm{dischg}}_{s,t}-{\mathrm{chg}}_{s,t}\geq \alpha_{\mathrm{TMR}}{\mathrm{gen}}_{t}^{\mathrm{ELY}}\beta^{\mathrm{ELY}}\forall t\in T$$

The annual TMR constraint, modeled by eq 3, enforces that the sum of annual generation from the eligible set of VRE resources (*g* ∈ TMR_*g*_) plus net battery injections (which will be negative due to roundtrip losses) must be equal to annual electrolyzer electricity consumption.

3 $$\sum_{g\in {\mathrm{TMR}}_{g}}\sum_{t\in T}{\mathrm{gen}}_{g,t}+\sum_{s\in {\mathrm{TMR}}_{b}}\sum_{t\in T}\left({\mathrm{dischg}}_{s,t}-{\mathrm{chg}}_{s,t}\right)=\sum_{t\in T}{\mathrm{gen}}_{t}^{\mathrm{ELY}}\beta^{\mathrm{ELY}}$$

As per eq 2, it is possible that the generation in some hours exceeds the electrolyzer power consumption to be matched, making the constraint nonbinding. Effectively, in these hours, contracted VRE resources are generating electricity in excess of the contractual requirement. Following the approach of Zeyen et al.,^16^ we included a constraint to limit the quantity of these “excess” electricity sales from contracted VRE resources (i.e., PPA resources) to the grid under the hourly TMR. Such a constraint is meant to discourage resources that would have been built for the grid from being designated as PPA resources in the optimal solution, thereby reducing model degeneracy. Practically, this constraint introduces a stronger operational relationship between PPA resources and the electrolyzer by ensuring that the majority of electricity generated by the VRE resources is contracted by the electrolyzer. In this way, it can be interpreted as a way to increase the degree of additionality of the PPA resources by minimizing the revenues obtained from electricity sales. Equation 4 restricts the quantity of electricity sales from contracted VREs to the grid at 120% (β = 0.2) of annual electrolyzer demand. The assumed value of β is based on assumptions in prior CEM studies evaluating TMR-based electricity procurement.^16,25^

4 $$\sum_{t\in T}\left(\sum_{g\in {\mathrm{TMR}}_{g}}{\mathrm{gen}}_{g,t}+\sum_{s\in {\mathrm{TMR}}_{b}}{\mathrm{dischg}}_{s,t}-{\mathrm{chg}}_{s,t}\right)\leq \left(1+\beta \right)\sum_{t\in T}{\mathrm{gen}}_{t}^{\mathrm{ELY}}\beta^{\mathrm{ELY}}$$

### 2.2 Case Study Description

We use a case study derived from generator and load conditions managed by the Electric Reliability Council of Texas (ERCOT) in 2021, where the existing generation capacity is dominated by natural gas (52.2 GW), followed by onshore wind (35.1 GW), solar (9.1 GW), coal (7.0 GW), and nuclear (5.0 GW) (see Table S5). Exogenous electricity demand data assumptions are the same as those used in our previous publication (see SI Figures 1–2 of Giovanniello et al.^12^), with peak demand of 75.7 GW and annual generation of 388.9 TWh, reflecting 2021 electricity demand patterns for the ERCOT grid.

**Figure 1:**
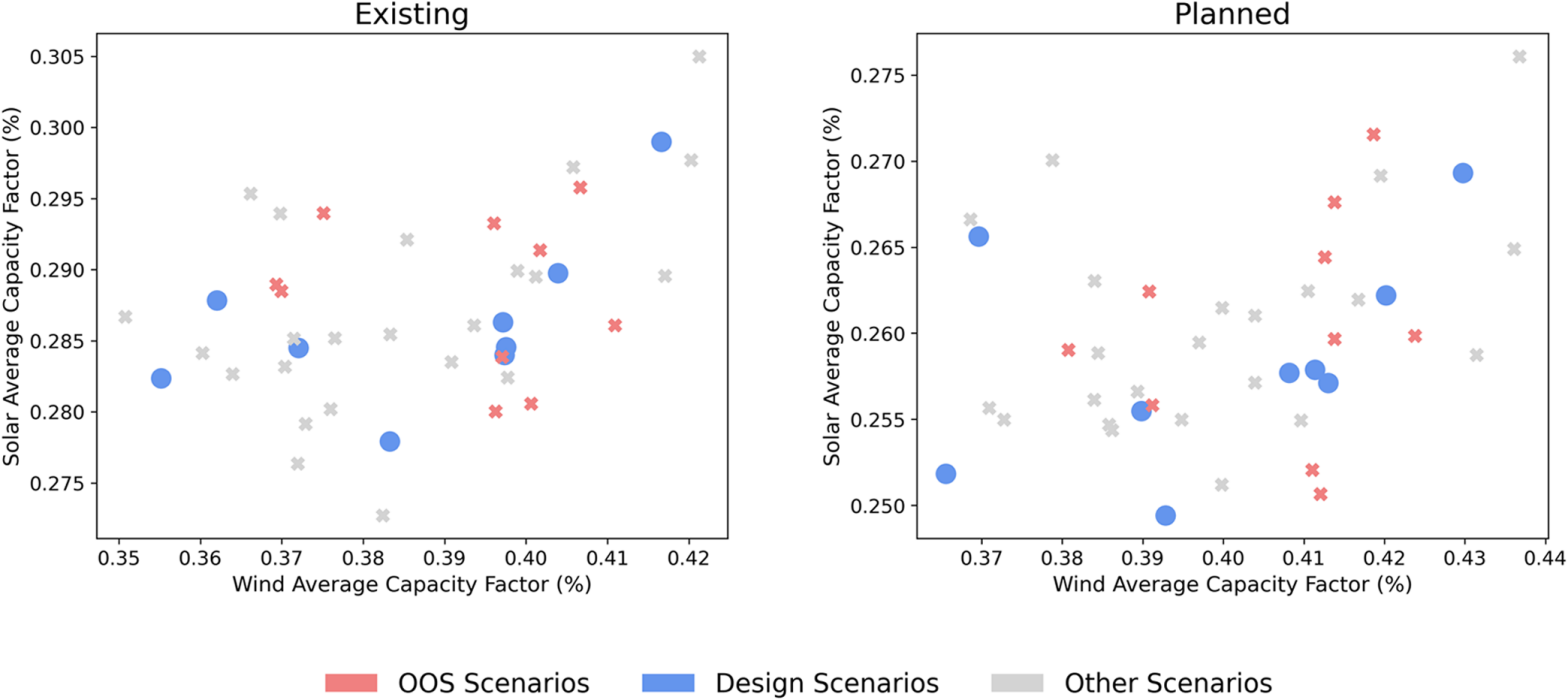
Scatter plot of annual averaged capacity factors of existing and planned wind (*x*-axis) and solar (y-axis) resources in the ERCOT hourly wind and solar generation profiles data set user here (1980–2021).^33^ The nine VRE profiles used for the in-sample analysis are shown as large blue circles; profiles for the out-of-sample analysis are red “X”; all remaining samples, which were used in the clustering analysis, are shown as gray “X”s. Figure S9 visualizes the same data organized by VRE resource type. Data from existing resource types are used to characterize the output of existing VRE capacity, while data from planned resources are used to characterize the output of new capacity additions in the model.

Our choice of H_2_ production as a case study is motivated by two factors. First, as noted earlier, clean electricity procurement requirements analogous to a TMR have been explicitly contemplated in policies supporting electrolytic H_2_ production in both the US and EU, making it a policy-relevant application. Second, electrolytic H_2_ production offers the potential for flexible electricity consumption when deployed alongside H_2_ storage, making it a particularly instructive case study for examining how demand flexibility shapes the cost and emission outcomes of alternative electricity procurement strategies.

To represent a consumer interested in clean electricity procurement, we model 1 GW of hourly H_2_ demand (18.4 tonnes of H_2_ per hour) to be met solely via electrolyzer-based H_2_ production paired with H_2_ storage. The modeled constant hourly H_2_ demand is intended to reflect consumption patterns of existing industrial H_2_ consumers, many of which are located in Texas. For context, the modeled H_2_ demand is representative of a medium- to large-scale ammonia production facility (440 tH_2_/day, which is equivalent to approximately 2500 tNH_3_/day). Flexible electrolyzer operation is allowed in all scenarios (see S1.1), which previous analysis confirmed is the most economic operating mode^8,12,16^ and is likely to be the preferred approach in practice. Our analysis is agnostic to the end-use industrial process consuming the produced H_2_, which, by definition of the baseload H_2_ supply requirement, is not affected by electrolyzer or electric grid operation patterns. Electrolyzer cost and performance assumptions are summarized in Table S3 and reflect those of a PEM electrolyzer,^30^ which has greater operational flexibility than commercially available alkaline electrolyzers.

### 2.3 Technology Assumptions

Investment and operating costs assumptions for new generation resources are from the 2022 NREL Annual Technology Baseline^31,32^ (Table S2). Table S3 summarizes investment and operating costs assumptions for electrolyzer and gaseous H_2_ storage, the same assumptions used in our previous study.^12^ Model runs with retirement and expansion of grid resource capacity allow specifically for retirement of existing natural gas and coal, and new construction of wind, solar, battery storage, as well as new natural gas generation capacity if needed. Nuclear, hydro, and biomass are not subject to retirement or new construction. The model includes two Li-ion battery sizing decision variables, power capacity and energy capacity, linked by a constraint that bounds the energy-to-power ratio between 0.15 and 12 h.

### 2.4 VRE Availability Data Inputs

Hourly solar and wind availability profiles for different weather scenarios are constructed using data from the ERCOT Hourly Wind and Solar Generation Profiles dataset.^33^ The dataset includes generation for solar and wind plants from 1980 to 2021. For plants built after 1980, historical generation profiles from 1980 are simulated based on spatially granular historical weather data. The dataset also includes simulated generation profiles for plants that were under construction in 2020.

We divided wind and solar resources into two groups, existing resources and planned resources, coresponding to sites where capacity could be added. For both resources, we created spatially aggregated capacity-weighted resource availability profiles by summing generation and dividing by total capacity for both existing and planned resources for each year. The result is four availability time series per weather scenario—one for existing and one for planned for both solar and wind—where the existing and planned profiles are used for existing and new build resources in the model, respectively (see S3.1 for details). Figure 1 highlights the interannual variation in annual average capacity factor for the four VRE resource types considered in the analysis, while Figures S10–S11 highlight the variation in hourly capacity factor data for each resource type.

Solving the stochastic model with 41 weather scenarios available in the ERCOT data set is computationally challenging with commercial optimization solvers such as Gurobi and the available computing resources (see S1.5) when also considering a granular representation of the existing generation fleet (40 existing generators). To address this, we applied k-means clustering to identify nine representative weather scenarios (blue markers in Figure 1; see section S3.1 for details), where each representative scenario is defined as the historical year closest to the centroid of its respective cluster. These nine weather scenarios are used in the in-sample analysis, which includes the nine deterministic model runs that each consider one weather scenario and a stochastic model run that considers all nine scenarios. Out-of-sample weather scenarios were selected by randomly sampling 10 of the remaining weather scenarios, which are highlighted with red markers in Figure 1.

**Table 1:** Description of Model Scenarios Evaluated and Their Corresponding Labels Used in Later Figures

**description of evaluated scenarios**	**scenario labels**
deterministic (D) model with annual (A) or hourly (H) TMR constraint with 100% compliance for different weather years (1–9)	Di-A for *i* = 1, ..., 9
	Di-H for *i* = 1, ..., 9
stochastic (S) model with annual (A) or hourly (H) TMR with 100% compliance	S–A, S–H
stochastic (S) model with hourly (H) TMR constraint serving at least a fraction of demand (*y*) and limits on H_2_ storage availability, equal to *x* hours of H_2_ demand	S–H–*y*–*x*L or S–H–*y* for *y* = 80%, 90%, *x* = 24, 48 h
stochastic (S) model with annual (A) or hourly (H) TMR constraint with an *x*% renewable portfolio standard (RPS) constraint applied to non-H_2_ demand	S–A-RPS*x* or S–H-RPS*x* for *x* = 60, 70, 80
stochastic (S) model with an *x*% renewable portfolio standard (RPS) constraint applied to all electricity demand (non-H_2_ and H_2_) and no TMR constraint on H_2_ demand	S-A-RPS*x*_all for *x* = 60, 70, 80

### 2.5 Scenarios Evaluated and Metrics of Interest

We assess the effects of annual and hourly TMRs under various scenarios of model setup, weather scenario, technology availability, and policy design, as summarized in Table 1. We quantify our findings in terms of impacts on model investments (power, electrolyzer, and H_2_ storage capacity), emissions, and cost.

Emission impacts are quantified as the difference in grid-level emissions associated with system-wide power generation in the case of H_2_ demand minus the emissions in the identical scenario without H_2_ demand, divided by total annual H_2_ production. This metric can be interpreted as the consequential emission impacts of H_2_ production with clean electricity procurement using TMRs. It should be noted that this metric does not account for the avoided emissions from displacing any existing fossil-based hydrogen production. Since these avoided emissions are independent of the procurement strategy employed, they do not affect comparisons across scenarios but should be considered when interpreting consequential grid emission estimates in absolute terms. In addition, our emissions metric only includes CO_2_ emissions from fuel combustion associated with coal- and NG-based electricity generation and does not account for upstream emissions related to fuel production, manufacturing, or non-CO_2_ greenhouse gas emissions. Incorporating these elements would affect the emission impacts of the various scenarios evaluated here (Table 1), though directional trends are likely to remain consistent given the predominance of fuel combustion emissions.

The economic viability of the H_2_ project is quantified using the LCOH, which is the annualized, all-in cost for the project developer per kilogram of H_2_ produced. LCOH considers the following costs: (a) fixed and variable costs of the electrolyzer, (b) fixed and variable cost of H_2_ storage and compression, (c) cost of electricity purchases (capacity and energy) to operate the electrolyzer and compressor, (d) fixed and variable costs of procured power generation and storage resources (referred to as power purchase agreement (PPA) resources from here on), and (e) revenue obtained from the sale of energy to the grid by PPA resources associated with the H_2_ project. LCOH represents the lowest selling price per unit of H_2_ that is required for the combined PPA and H_2_ generation and storage assets to break even.

The robustness of capacity decisions under hourly TMR is assessed by exposing them to weather scenarios not considered during system sizing and measuring the extent to which they satisfy key operational constraints, including hourly supply demand balance for H_2_ and electricity and the TMR constraint (eqs 2–3). To do so, we fix the capacity decisions (investments and retirements) obtained from the deterministic and stochastic models and evaluate the optimal operation of the resulting electricity–H_2_ system under alternative, out-of-sample weather scenarios. Robustness is quantified by the optimal value of slack variables in the key operational constraints that are subsequently penalized in the objective function to minimize constraint violation. The optimal value of the slack variables measures the degree to which in-sample capacity decisions fail to maintain operational feasibility under previously unseen weather scenarios. The optimal value of all slack variables in the CEM is zero by design.

We visualize the slack variable for the hourly TMR constraint in terms of (a) the *number of hours where time-matching is unmet* and (b) *the share of hydrogen production that is unmatched in those hours.* The first metric corresponds to the number of hours in the year where the slack variable is nonzero for the TMR constraint (see S1.3). The second metric is the ratio of the sum of TMR slack variables (units of MWh) in hours with unmatched H_2_ production divided by the total electricity demand for H_2_ production in those hours.

To quantify the additionality of VRE resources added in the presence of a TMR constraint, we calculated the revenues per MW for PPA and grid VRE resources. For PPA resources, revenues accrue from the sale of energy to the grid, as well as the TMR constraint, while for grid VRE resources, revenues accrue from the sale of energy and capacity to the grid and from contributing to the RPS constraint if modeled.

## 3 Results

### 3.1 Deterministic vs Stochastic Model Results

The optimal sizing of PPA resources, electrolyzer capacity, and H_2_ storage for the deterministic models is highly sensitive to weather scenarios, as shown in Figure 2. This sensitivity is most pronounced with annual matching, where a single VRE resource typically dominates the PPA capacity mix, depending on scenario-specific resource availability patterns. Under hourly matching, weather scenarios have less influence on PPA composition because meeting the more stringent constraint requires relying on both resources.

**Figure 2:**
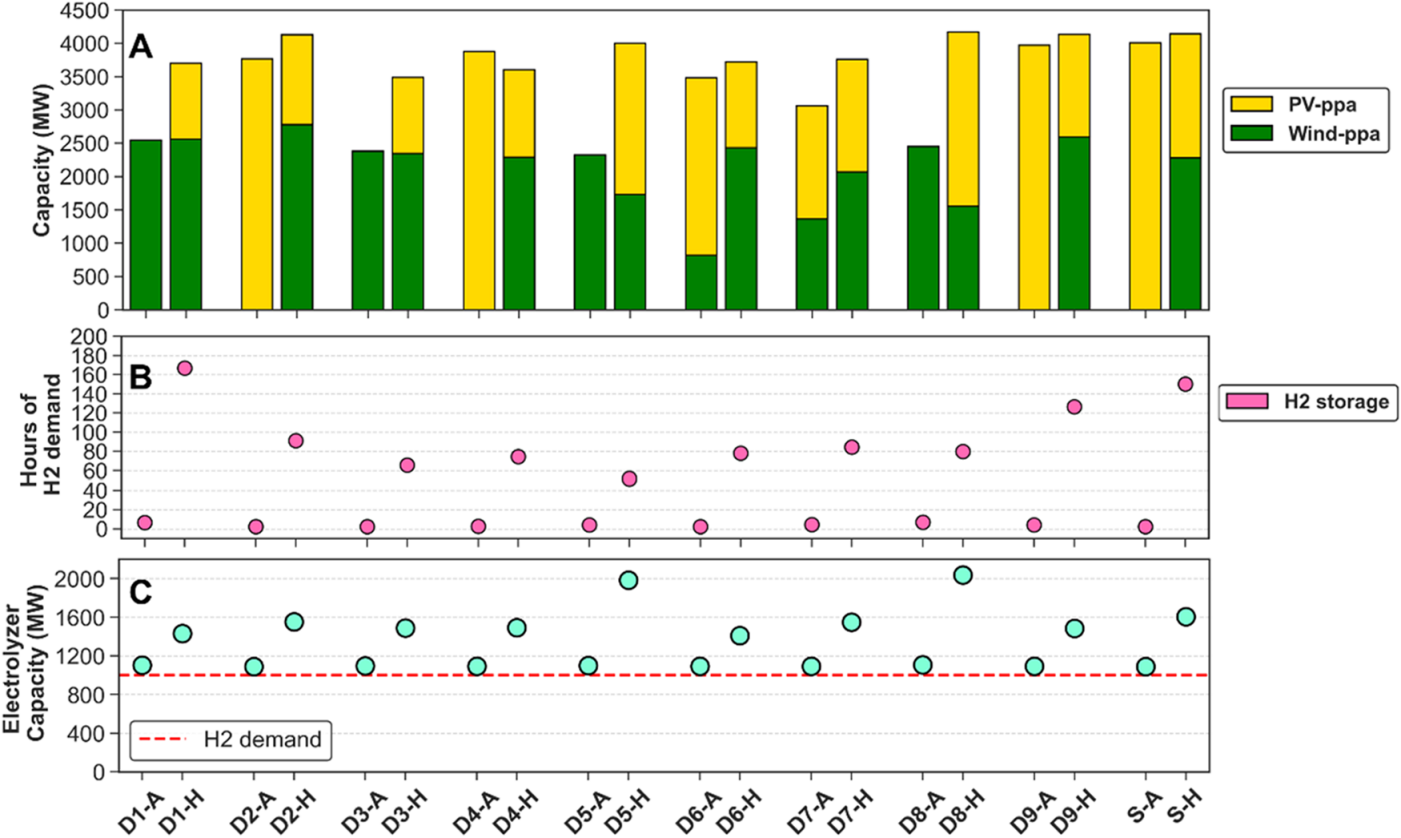
PPA resources and H_2_ project design for the stochastic and deterministic models under annual and hourly time matching requirements. PPA VRE and battery power capacity additions (A), energy storage capacity by storage technology (B), and installed electrolyzer capacity (C). The stochastic model (labeled “S-A” or “S–H,” corresponding to annual or hourly TMR) co-optimizes design over nine weather scenarios, while the deterministic model is solved for each of the nine weather scenarios (labeled “DX-A” or “DX-H” where X is the weather scenario). H_2_ storage capacity is reported in terms of hours of H_2_ demand, which is calculated by dividing the H_2_ storage capacity by the baseload H_2_ demand (18.4 tonnes/hour). . No PPA battery storage is deployed across the modeled scenarios.

**Figure 3:**
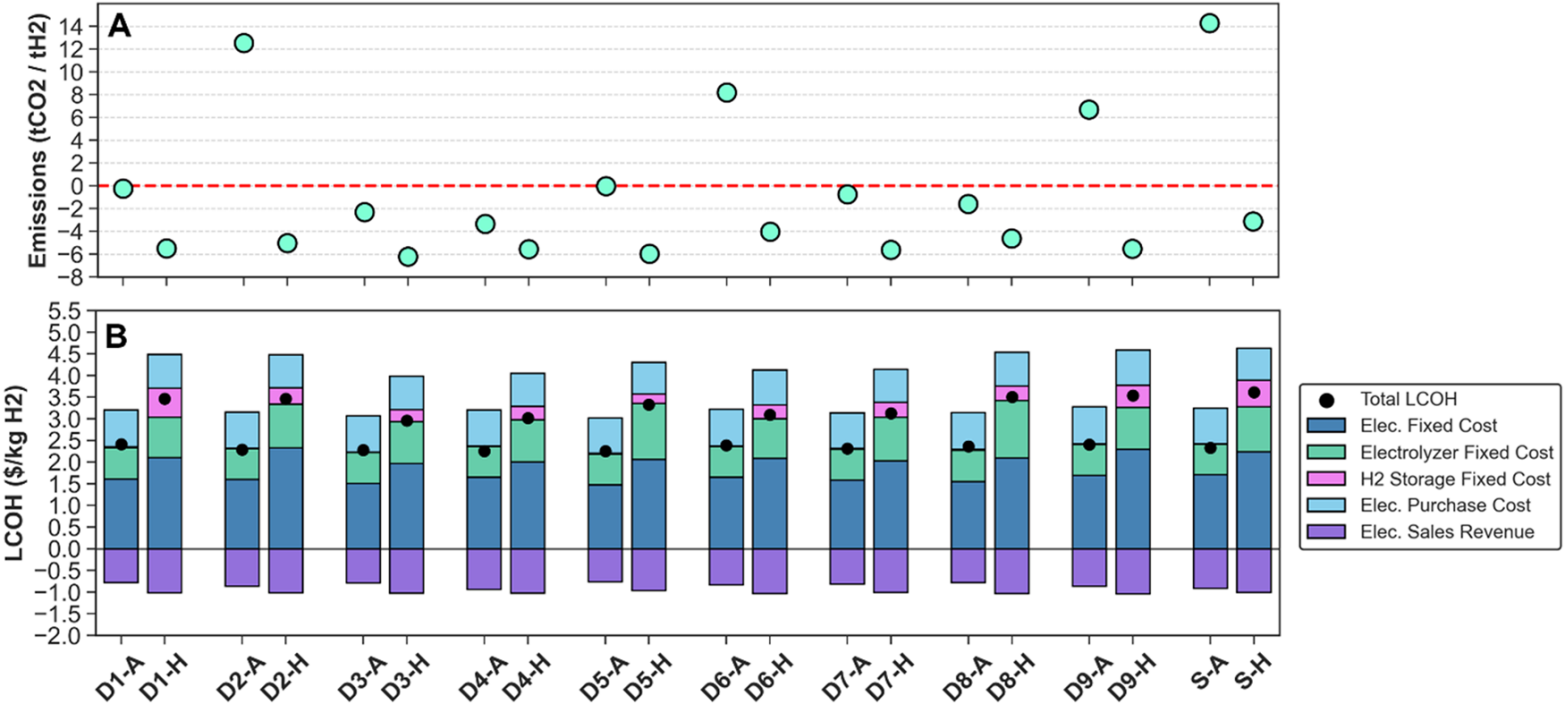
(A) Consequential emissions intensity of H_2_ production under different model cases, corresponding to different TMR (annual, hourly) and model configurations, stochastic and deterministic, under nine different weather scenarios. (B) Levelized cost of H_2_ production associated with deterministic and stochastic model runs under annual and hourly TMR. Model configuration nomenclature as defined in Table 1 and Figure 1 caption.

Electrolyzer and H_2_ storage capacity decisions are more sensitive to weather scenarios under hourly matching than annual matching, where optimal electrolyzer capacity is between 1.4 and 2 times baseload H_2_ demand, while H_2_ storage capacity varies between 52 and 167 h of exogenous H_2_ demand. We find that it is economical to oversize the electrolyzer relative to H_2_ demand and deploy relatively less expensive H_2_ storage rather than battery storage to satisfy hourly TMR, as in previous modeling studies.^12,16^ This approach allows the electrolyzer to adjust its power consumption in response to hourly fluctuations in VRE generation from PPA resources, while H_2_ storage buffers supply to meet the baseload H_2_ demand. In contrast, under annual matching, operating the electrolyzer near baseload operation is generally more economical, as indicated by lower H_2_ storage capacity (2–7 h of H_2_ demand, Figure 2B) and electrolyzer capacity (1.1 times baseload H_2_ demand, Figure 2C). The difference in electrolyzer dispatch between hourly and annual matching cases is also evident in the average hourly incremental generation profiles compared to the baseline (no-H_2_) model scenario. (Figure S1 vs Figure S2).

The stochastic model preserves the key differences in asset sizing between annual and hourly matching observed in the deterministic model. Under hourly matching, the stochastic model favors deploying more H_2_ storage, oversizing the electrolyzer, and combining wind and solar PPA capacities. Notably, the stochastic model’s optimal H_2_ storage and electrolyzer capacities approach the highest values observed across the individual deterministic weather scenarios. This suggests that increased H_2_ storage and electrolyzer capacity, which collectively represent a form of electricity demand flexibility, hedge against both intra-annual and interannual weather variability in satisfying the hourly TMR constraint.

Figure 3A summarizes the emissions impact of electricity-based H_2_ production under annual versus hourly matching. Under annual matching, consequential emissions span a wide range, from near-zero to 14 tCO_2_/tH_2_. In contrast, hourly matching consistently yields negative consequently emissions, indicating that a grid operating with H_2_ demand and an hourly TMR constraint produces lower emissions than the baseline. For context, attributional life cycle greenhouse gas emissions from natural gas-based H_2_ production without CO_2_ capture are approximately 10 tCO_2_eq/tH_2_.^34,35^ Among the nine deterministic model cases with annual matching, six achieve near-zero or negative emissions. It should be noted, however, that hourly time-matching requirements, by necessitating larger electrolyzer and battery capacities, may increase embodied manufacturing emissions not captured in our emissions metric, potentially offsetting some of the grid-level emissions reductions estimatedhere.

The emission outcomes seen in Figure 3A result from changes in capacity deployment and utilization induced by the TMR, as summarized in Figure S4. Under annual matching, positive emissions occur when solar dominates the PPA capacity mix. This is because PPA solar displaces grid solar deployed in the no-H_2_ baseline case and because during hours when the PPA solar is not generating, additional gas generation is needed (Figure S1) to meet H_2_ production’s near-baseload electricity demand, given the limited energy storage capacity (Figure 2B). Conversely, grid VRE and gas generation are largely unaffected when sufficient wind capacity is deployed to meet the annual matching constraint, resulting in a near-zero emissions impact. The more stringent hourly matching constraint leads to a mix of wind and solar capacity and flexible electrolyzer operation, all of which mitigate the mismatch between the incremental supply and demand without inducing additional gas generation. Moreover, the greater build-out of wind and solar capacity relative to incremental electricity demand under hourly matching (Figure 1A) leads to higher (6–13%) VRE curtailment as compared to near-zero curtailment in the annual matching cases (Figure S3).

These findings align with prior assessments indicating that annual matching with grid-PPA competition can yield higher emissions than hourly matching.^12,13,16^ However, here we demonstrate that generalizing from deterministic assessments requires caution as emissions outcomes under annual matching are highly sensitive to the selected weather scenario. This underscores the need to consider interannual weather variation in the assessment of long-term emission impacts of clean electricity procurement strategies based on hourly matching.

Figure 3A shows that, compared to deterministic model outcomes across weather scenarios, the stochastic model estimates higher emissions under annual matching and smaller emission reductions under hourly matching. Regarding resource deployment, the stochastic model retains some coal capacity under both TMR approaches, primarily to meet the resource adequacy requirement (eq S1), whereas deterministic cases virtually eliminate all coal capacity.

The lower emissions impact of hourly matching versus annual matching comes with a cost premium (Figure 3B) that ranges between $0.68 and 1.18/kg H_2_ across the different weather scenarios with the deterministic model and $1.29/kg H_2_ under the stochastic model. This highlights how a deterministic model may underestimate the cost premium associated with the hourly matching. For context, the LCOH of H_2_ production via natural gas-based H_2_ production in the U.S. context, with and without CO_2_ capture, is estimated to be around $1/kg and $1.5–2/kg, respectively.^34–36^ Hourly matching is more expensive because it requires more PPA VRE resources, as well as greater electrolyzer and H_2_ storage capacity (Figure 2).

### 3.2 Out-of-Sample Analysis

Figure 4 shows that stochastic modeling produces a solution that is more robust at meeting an hourly TMR when exposed to out-of-sample weather scenarios than does the deterministic model. Of the 10 out-of-sample weather scenarios tested, the stochastic model solution is able to accommodate all without having to relax the hourly TMR constraint, whereas the deterministic model design solutions require relaxing the constraint for 48 of the 90 runs (9 deterministic cases x 10 out-of-sample weather scenarios per case). Across the cases, the number of hours in which the hourly time-matching requirement was not fully satisfied ranges from 0 to 744. The average share of electricity demand for H_2_ production that does not match during those hours is calculated for each case, with a median of 16% across cases and a maximum of 51%.

**Figure 4:**
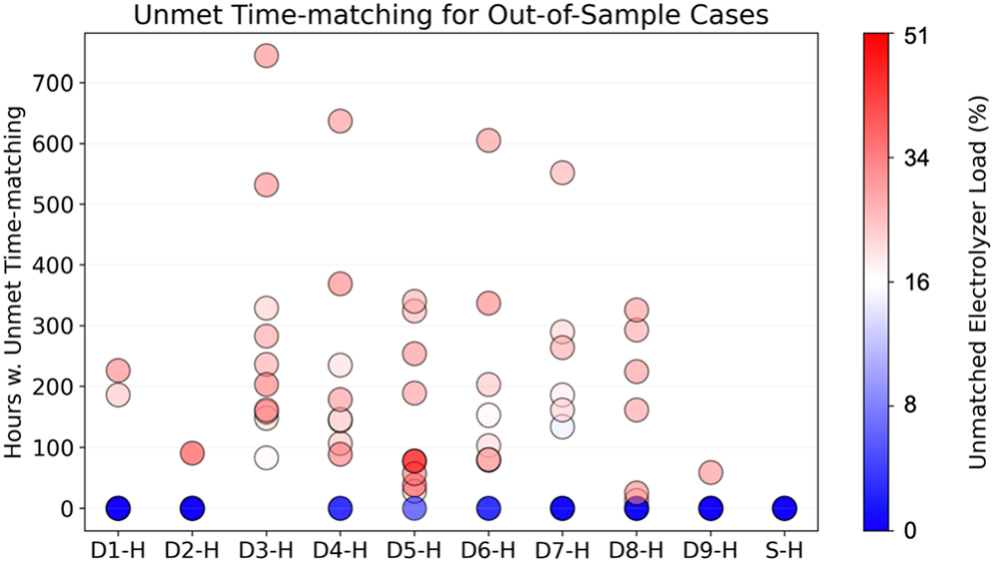
Unmet time-matching for out-of-sample cases for stochastic and deterministic model design solutions under an hourly time-matching requirement. Marker position corresponds to the left axis, which shows the number of hours in an out-of-sample case in which the full time-matching requirement was not fully satisfied. Unmet time-matching is enabled by utilizing a slack variable that permits electricity used by the electrolyzer to be greater than the generated electricity by PPA resources in that hour. The color of the markers indicates the average share of the time-matching requirement that was not met during hours when the requirement was not fully satisfied (i.e., “Unmatched Electrolyzer Load (%)”), which is calculated as the sum of TMR slack variables (corresponding to MWh) in hours with unmatched H_2_ production divided by the total electricity demand for H_2_ production in those hours.

The robustness of the stochastic solution to out-of-sample weather scenarios is in part driven by the deployment of hydrogen storage (Figure 2); however, this level of H_2_ storage build-out may exceed real-world availability (see the next section). We replicated the out-of-sample analysis described above for stochastic cases where the H_2_ storage build-out is constrained (Figure 5) to test whether the stochastic solution remains robust when H_2_ storage is limited. Somewhat surprisingly, we find that the stochastic model solutions remain robust to out-of-sample weather years, with no slack utilization across the cases evaluated. This suggests that increasing the capacity of wind and solar resources in a balanced portfolio maintained the robustness of the solution, compensating for constraints on H_2_ storage capacity.

**Figure 5:**
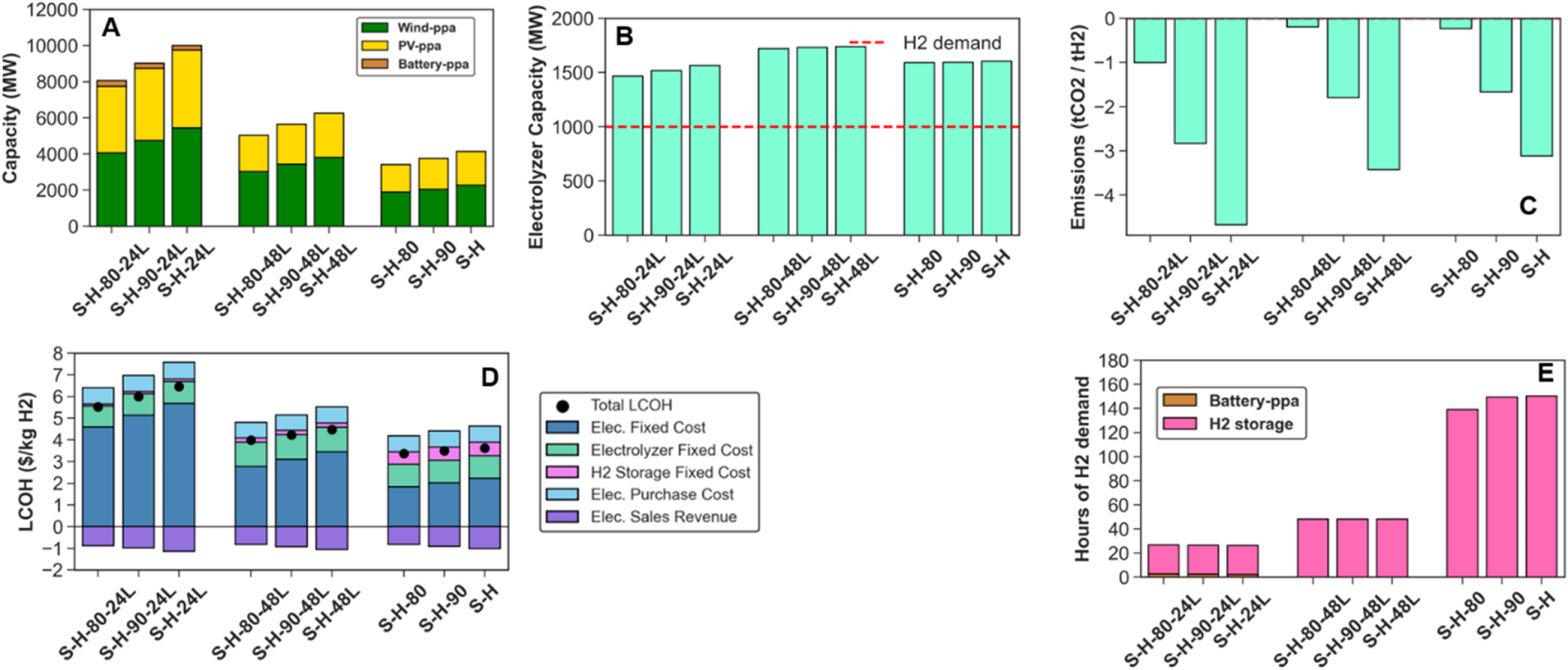
Impact of limiting H_2_ storage capacity and degree of compliance with the hourly matching constraint on the stochastic model outcomes: (A) PPA capacity mix, (B) electrolyzer capacity, (C) consequential emissions, (D) levelized cost of hydrogen (LCOH), and (E) energy storage capacity. Results based on a stochastic model with an hourly time matching requirement. S–H = Stochastic model with total hourly matching; S–H-X = Stochastic model with hourly matching with X percent (80,90) compliance of the constraint; S–H-X-YL = Stochastic model with hourly matching with X percent compliance and limit on H_2_ storage capacity equal to Y hours of H_2_ demand (Y = 24, 48). S–H–YL= Stochastic model with hourly matching with 100% compliance and limit on H_2_ storage capacity equal to Y hours of H_2_ demand (Y = 24, 48). See Table 1 for further explanation of scenario labels.

### 3.3 Impacts of Reducing TMR Compliance Stringency and Limiting H_**2**_ Storage Capacity

We use the stochastic model to investigate two practical considerations for implementing hourly matching in electricity-based H_2_ production. First, we explore the impact of reducing the compliance threshold (α_*TMR*_ in eq 2) for hourly matching to less than 100% (80% or 90%), because strict hourly matching may be prohibitively expensive for some applications. Second, we limit the capacity of above-ground H_2_ storage, which may result from implementation constraints (e.g., land use^37^) arising from the low volumetric energy density and flammability of hydrogen. For reference, the amount of H_2_ storage capacity installed for the stochastic model with hourly TMR (2,769 tonnes, see Figure 1B) is similar to the size of the largest operational underground H_2_ storage facility.^38^

As expected, reducing compliance stringecy to 80% or 90% decreases PPA and energy storage capacity investments (Figure 5A,E) and, consequently, the cost of H_2_ production (Figure 5D). However, the cost savings are relatively modest—6.8% and 3.4% for 80% and 90% compliance, respectively (Figure 5D)—when compared to the 35% cost increase of hourly versus annual matching in the stochastic model. The reduced PPA capacity under partial compliance also diminishes system-wide emissions reductions, with 80% compliance achieving near-zero emissions impact rather than negative emissions (Figure 5C). These results have implications for procurement strategy: if consumers prioritizing hourly matching seek primarily to minimize their own emissions impact rather than achieve system-wide reductions, then partial compliance (e.g., 80% in the modeled case) may offer a more practical and cost-effective approach than hourly matching with 100% compliance.

Compared to varying compliance thresholds, restricting H_2_ storage availability has a more substantial impact on PPA and H_2_ asset design under hourly matching (Figure 5). When H_2_ storage is constrained to 24 or 48 hours of rated H_2_ demand, achieving 100% hourly matching compliance requires substantially more PPA VRE capacity—approximately 50% and 150% increases (Figure 5A), respectively—along with additional electrolyzer capacity and battery energy storage in some cases. These capacity additions translate to LCOH increases of 78% and 24% relative to the unconstrained storage case (Figure 5D) for the 24- and 48-hour storage limit scenarios, respectively. In addition, relaxing the compliance threshold offers greater cost savings when storage availability is limited. For example, reducing hourly matching compliance from 100% to 90% with a 24-hour H_2_ storage limit lowers costs by 7% as compared to 3.3% under the unconstrained storage availability case. Finally, limited H_2_ storage availability also reduces the degree of compliance needed to achieve near-zero emissions impact (Figure 5C).

### 3.4 Impact of an RPS Policy

Projects subject to temporal matching requirements will not exist in isolation but within existing policy frameworks governing electricity-system transitions. Here, we examine the interaction between TMR for new electricity-based H_2_ production and RPS constraints on existing electricity demand, which is a common decarbonization policy employed across many regions in the United States and around the world.

A binding RPS constraint on non-H_2_ electricity demand mitigates the displacement of grid VRE capacity that would otherwise occur when adopting new demand with an annual TMR (see Figure S5). In effect, an RPS constraint reduces competition between VRE capacity deployed to meet the TMR constraint and VRE capacity deployed solely based on the economics of electricity supply to the grid. Consequently, the emissions impacts of annual time-matching under a binding RPS constraint for non-H_2_ electricity demand are low or negative and largely similar to those of hourly matching (Figure 6A). An RPS constraint has minimal impact on emissions under an hourly requirement (Figure 6A), which does not lead to displacement of grid VRE capacity even in the absence of an RPS constraint (Figure S5). RPS requirements have a minimal impact on H_2_ asset deployment (Figure S6), with no change under hourly matching and only slight increases in electrolyzer and H_2_ storage capacity under annual matching to capitalize on increased electricity price volatility from higher VRE penetration.

A binding RPS requirement on non-H_2_ electricity demand suppresses energy prices—specifically, the shadow price of the hourly electricity supply demand balance constraint (Figure S7)—which affects LCOH through two countervailing mechanisms: (a) it reduces electricity purchase costs for H_2_ production and storage, and (b) it reduces revenue from electricity sales by PPA VRE resources. These opposing effects largely offset each other, resulting in minimal net impact on LCOH as seen in Figure 6B.

**Figure 6:**
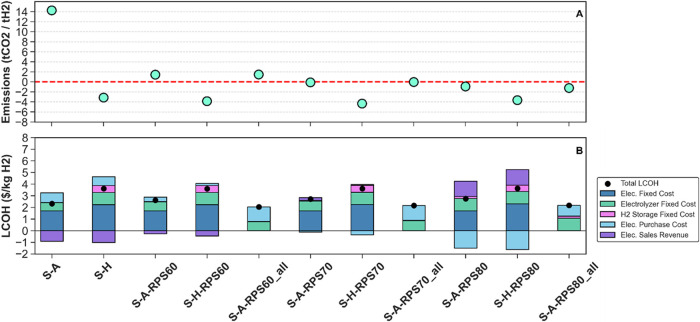
Consequential emissions (A) and levelized cost of hydrogen (B) from stochastic model runs with varying renewable portfolio standard (RPS) requirements for non-H_2_ electricity demand (no RPS, 60%, 70%, and 80%). ″RPS 60″ indicates annual grid VRE generation has to be atleast 60% of non-H_2_ electricity demand and so on. ″RPSX_all″ includes electrolyzer consumption in the RPS constraint and without a time-matching requirement. S-A/S–H: annual/hourly time-matching. S-A-RPSXX/S–H-RPSXX: annual/hourly time-matching for electrolyzer with XX% RPS for non-H_2_ demand. Note: For “RPSX_all” scenarios, electricity purchase cost by electrolyzer and compressor includes (a) energy price, (b) capacity price, and (c) RPS price. Prices are summarized in Figure S7. See Table 1 for further explanation of scenario labels.

The impact of an RPS requirement on TMR prices, referring to the shadow price of the TMR constraint, is more substantial, particularly under annual matching (Figure S7). TMR prices can be interpreted as the cost of energy attribute certificates (EACs) to be purchased by the H_2_ producer from the PPA VRE generators as part of their executed PPA. TMR prices increase under binding RPS constraints to compensate PPA VRE resources for reduced energy revenues caused by wholesale energy price suppression. Under annual matching, TMR prices increase from $14/MWh without an RPS to $26/MWh, $36/MWh, and $55/MWh under RPS60, RPS70, and RPS80, respectively.

We also evaluate an alternative scenario where H_2_ producer’s electricity demand is included within the binding RPS constraint rather than subject to separate matching requirements (RPSXX_all scenarios in Figure 6) . This approach yields near-zero consequential emissions outcomes but at a lower cost to the H_2_ producer compared to annual and hourly time-matching. The cost advantage versus annual matching arises because H_2_ producers only need to contract for VRE supply equal to the RPS requirement (less than 100%), rather than match 100% of their consumption. For example, under RPS60, a H_2_ producer consuming 54.3 kWh/kg H_2_ must procure EACs associated with the RPS for only 60% of electricity consumption or 32.58 kWh/kg H_2_. With an RPS price of $25.7/MWh (shadow price of RPS constraint, reported in Figure S7), this translates into a cost of $0.84/kg H_2_. In contrast, the annual TMR price under RPS60 is approximately $26/MWh (Figure S7), resulting in a TMR EAC cost of $1.4/kg H_2_ (54.3 kWh/kg x $26/MWh), which is 67% higher because annual matching requires EACs for 100% of consumption.

Figure 7 compares revenue sources for grid and PPA VRE resources across several RPS requirements (60%, 70%, and 80%) and a no-RPS scenario, expressed on a dollar-per-MW basis. Here, revenues from the various constraints are estimated based on the corresponding shadow prices, summarized in Figures S7–S8. Under the modeled CEM framework, revenues are necessarily greater than or equal to costs for all installed resources,^39,40^ which is substantiated in Figure 7. Under hourly time-matching, PPA resources derive the majority of their revenue from the TMR constraint across all scenarios, reflecting a strong causal link between the TMR constraint and resource deployment and implying strong additionality. Under annual time-matching without an RPS constraint on non-H_2_ demand, PPA resources (solar) earn a smaller share of revenue from the TMR, suggesting that resource deployment is less strongly tied to the constraint, indicating weaker additionality relative to hourly time-matching. When a binding RPS constraint on non-H_2_ demand is introduced, however, annual time-matching achieves comparably strong additionality—PPA resource revenue is predominantly sourced from the TMR constraint, similar to the pattern observed under hourly time-matching. This suggests that annual TMR coupled with a binding RPS policy on non-H_2_ demand offers similar levels of additionality as hourly time-matching but at a lower cost to the H_2_ producer (Figure 6B).

**Figure 7:**
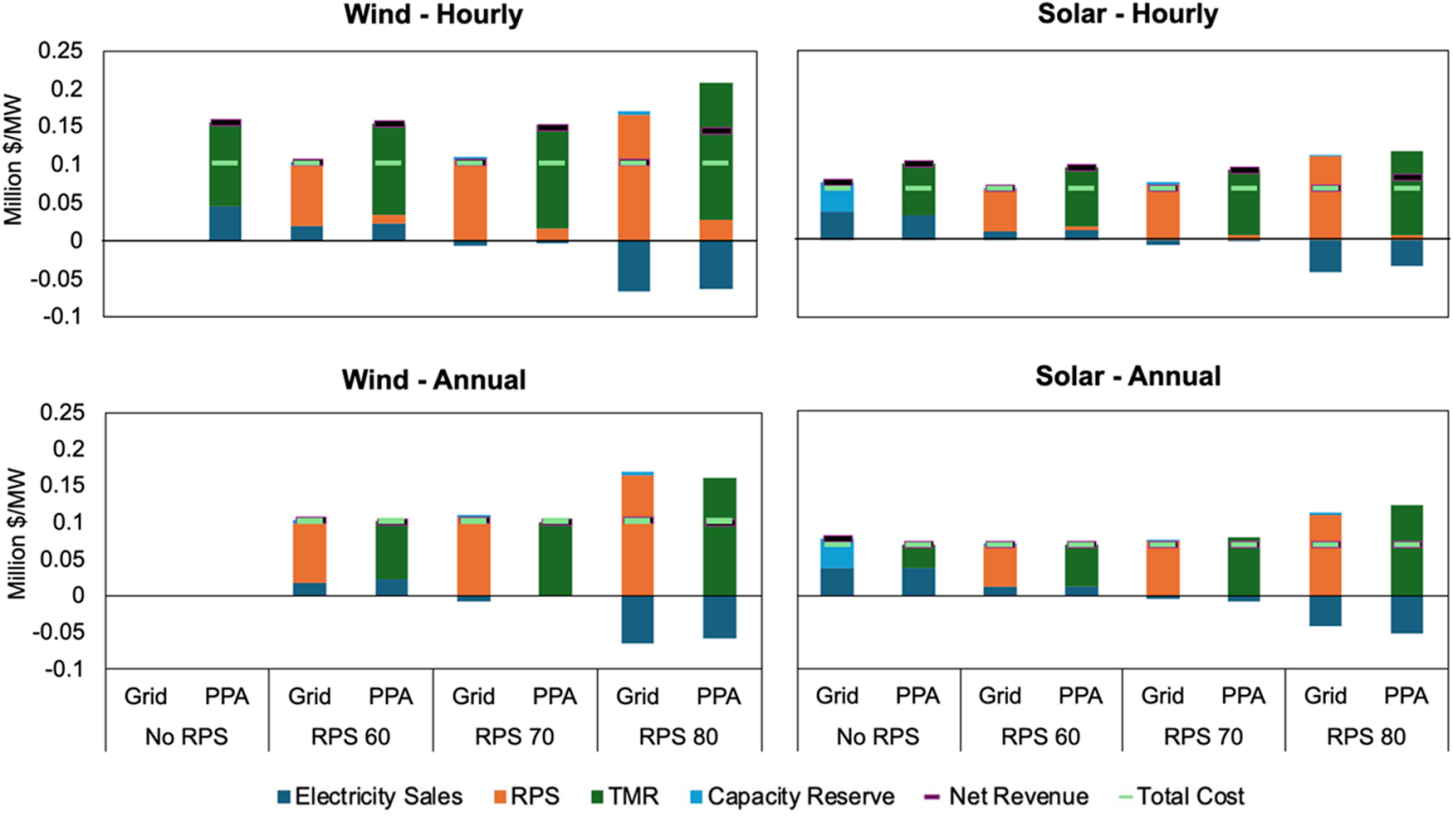
Comparison of costs and revenues for newly installed grid and PPA VRE resources under different scenarios with and without an RPS requirement on non-H_2_ electricity demand. “Grid” resources are new VRE that serve the grid generally, whereas “PPA” resources are those built to meet the time-matching requirement of the H_2_ project. “Electricity sales” is the revenue earned from grid injections by both PPA and grid resources. “RPS” revenues are earned by only grid resources for selling RPS energy attribute certificates. “TMR” revenues are earned only by PPA resources for satisfying the time-matching requirement. “Capacity Reserve” revenues are earned only by grid resources for contributing to the capacity reserve requirement (eq S1). “Net Revenue” sums all revenue sources. “Total Cost”, reflects the investment and operating cost of VRE capacity as per cost assumptions summarized in Table S2.

Under a binding RPS constraint, grid resources earn the majority of their revenue from RPS payments. Without an RPS, their revenue comes from a mix of capacity reserve payments and electricity sales. Electricity sales comprise a minor share of the revenues for all VRE resources. As the stringency of the RPS constraint increases, electricity sales generate increasingly negative revenues because renewable resources must be generated during periods with negative electricity prices to comply with both RPS and TMR obligations. Capacity reserve payments constitute a minor share of the revenue stack for grid resources under a binding RPS, reflecting the declining capacity value of VRE resources with increasing deployment.^41^

The revenue of PPA resources under an hourly TMR exceeds their costs due to the cap on excess energy sales to the grid (eq 4). This additional ″rent″ earned by PPA resources indicates that without the excess energy sales cap, deploying more PPA resources would be profitable. This explanation is corroborated by the observation that PPA resources do not earn rent under the annual time-matching requirement, where excess energy sales are prohibited by design (eq 3). As noted by others,^16,25^ the excess energy sales cap constraint for hourly TMR is a modeling construct that allows for distinguishing between resources deployed to meet the TMR constraint and those deployed purely on the basis of power grid economics.^16,25^

## 4 Discussion

We investigated the impact of interannual weather variability on the technology mix, costs, and emissions of electrolyzer projects subject to annual and hourly time-matching requirements using a CEM that accounts for competition between VRE resources for H_2_ production and grid applications. We also tested the impacts of (a) relaxing the hourly TMR constraint, (b) limiting H_2_ storage availability, and (c) enforcing an RPS requirement.

Our results reaffirm prior findings that annual TMR yields lower costs but higher emissions than hourly TMR^5,12,13,16,25^ and additionally show that annual time-matching emissions outcomes are highly sensitive to the assumed weather scenario. This underscores the importance of incorporating interannual weather variability, for example, through the stochastic modeling approach evaluated here, when assessing system design and long-term emissions impacts of alternative TMRs. The stochastic model preserves the qualitative differences in system design, costs, and emissions impacts between annual and hourly matching observed in deterministic approaches, while better accommodating interannual VRE variations, as evidenced by stochastic designs yielding zero periods of unmatched H_2_ production in the out-of-sample dispatch analysis. However, the difference in cost between hourly and annual TMR is greater in the stochastic model, suggesting that deterministic approaches may underestimate the cost premium of hourly matching, while also likely generating solutions that could fail to completely match the electrolyzer’s consumption with procured clean electricity in practice.

We also examined practical implementation considerations for hourly matching, including partial compliance (matching less than 100% of hourly demand) and limited availability of H_2_ storage that enables flexible operation. Partial compliance may appeal to consumers seeking near-zero rather than deeply negative consequential emissions at a reduced cost, particularly when H_2_ storage is constrained. Achieving full compliance would likely require spot markets for hourly time-matched EACs, where consumers can purchase shortfalls not covered by their long-term procurement contracts. Such markets could exhibit highly skewed pricing due to the coincidental nature of regional VRE supply: a few high-price hours during periods of limited VRE availability, accompanied by many low-price hours when VRE is abundant. Figure S8 illustrates this skewed distribution of hourly TMR prices across various stochastic model runs. This finding is consistent with empirical evidence from the European EAC market, where Scholta and Blaschke^42^ document increased instances of VRE demand exceeding VRE supply during nighttime hours compared to daytime hours.

Limited H_2_ storage availability, which constrains flexible electrolyzer operation, increases H_2_ production costs by 24–78% across the evaluated scenarios. More broadly, this underscores the critical role of demand flexibility in reducing VRE capacity requirements and costs under hourly matching. For baseload demands such as current data center operations,^43,44^ hourly matching may prove impractical without measures to improve demand flexibility.

Binding RPS requirements on existing electricity demand substantially diminish the incremental emissions benefits of hourly versus annual matching, because RPS constraints provide dedicated revenue streams for grid-oriented VRE deployment that effectively decouple it from competition with PPA VRE capacity. This reduced competition mitigates incremental use of fossil generation seen with annual matching without any RPS constraints on non-H_2_ electricity demands. In addition, we show that incorporating the new electricity demand directly into binding RPS constraints—rather than imposing separate time-matching requirements—achieves near-zero consequential emissions outcomes but at a lower overall cost to the consumer. This finding suggests that in regions with binding renewable energy targets or equivalent grid decarbonization policies, temporal matching-based clean electricity procurement may be less attractive as an emissions reduction strategy compared to participating in existing regulation-driven markets.

Future research could expand the stochastic modeling framework employed here to assess how transmission constraints and restrictions on the spatial boundary for VRE procurement impact the PPA system design, cost, and emissions outcomes of hourly versus annual matching. Prior work using deterministic demand-centric modeling approaches suggests that transmission constraints could affect grid dispatch and, therefore, the emissions impacts of hourly matching.^11^ Our analysis also did not consider spatial heterogeneity in VRE resource quality and grid integration costs, which could influence the relative competitiveness of different resources for the voluntary clean electricity procurement. Another important aspect not evaluated here is how increasing the adoption of time-matching as a clean electricity procurement strategy affects the cost of electricity supply to other consumers. The stochastic modeling framework employed here can also be extended to quantify regional variations in the impacts of TMR-based clean electricity procurement strategies. For instance, a recent study employing a single weather-scenario CEM framework found substantial regional variation in the emissions and cost impacts of hourly time-matching-based VRE procurement.^26^

Although this study demonstrates the importance of accounting for interannual weather variability using historical data, future work should examine how projected changes in VRE resource availability as a result of climate change and long-term system evolution—including the multiyear build-out of hydrogen infrastructure—affect the cost and emissions outcomes of alternative clean electricity procurement strategies. Finally, while our study has examined system impacts of voluntary procurement by a single consumer type, real-world markets involve consumers with varying demand patterns, flexibility potential, and preferences for clean electricity procurement. Decentralized modeling approaches that capture individual consumer consumption patterns, procurement decisions, and risk preferences could therefore provide insights into likely market outcomes under large-scale adoption of voluntary clean electricity procurement strategies.

## Supplementary Material



## Notes

Additional results for evaluated scenarios; summary of modeling approach, input assumptions, and implementation details (DOCX)
