# Supplementary material for: Emissions and Cost Trade-Offs of Time-Matched Clean Electricity Procurement under Interannual Weather Variability: A Case Study of Hydrogen Production
Source: Environ Sci Technol. 2026 Jul 6;60(28):19854–65. doi: 10.1021/acs.est.6c00988 (PMC13394394; doi:10.1021/acs.est.6c00988)
Supplement: Supplementary file 1 [file es6c00988_si_001.docx]

**Supporting information for**

**Emissions and cost tradeoffs of time-matched clean electricity procurement under inter-annual weather variability – case study of hydrogen production**

Michael A. Giovanniello^1^, Dharik S. Mallapragada^2^*

1. MIT Energy Initiative, Massachusetts Institute of Technology, Cambridge, Massachusetts 02139, United States

2. Chemical and Biomolecular Engineering Department, Tandon School of Engineering, New York University, Brooklyn, New York 11201, United States

*Correspondence: Dharik S. Mallapragada

**Email**: dharik.mallapragada@nyu.edu

**Summary**: This supporting information contains 19 pages, 11 figures, 6 tables, and 2 equations.

# S1. Modeling approach

## S1.1. Capacity expansion model overview

Table S 1 summarizes key constraints in the capacity expansion model for system-level and project-specific operation and their relation to investment decisions.

Table S 1. Summary of constraints associated with system and technology operation in the DOLPHYN CEM used in this study^23,24^. Further description of the specific policy constraints of interest to the model are provided in the supplementary information section S1.1. PPA = Power Purchase Agreement. “PPA generators” refers to those generators contracted by H_2_ production to satisfy the time-matching requirement constraint.

| Constraint Scope | Constraint Type |
| --- | --- |
| System-level constraints | - Supply-demand balance (hourly) - Capacity reserve margin constraint ensuring resource adequacy (hourly) - Renewable energy share requirements (annual) - Time-matching requirements (TMR) associated with PPA generators to serve electricity demand for H2 production (hourly or annual) |
| Thermal generators | - Power output must be less than available capacity at each time step - Minimum stable power output for each time step - Linearized unit commitment - Ramp rate limits (up & down) |
| Renewable Energy Generators (VRE, Hydro) | - Capacity constraint with time-dependent availability factor at each time step - Minimum production rate (Hydro only) at each time step - Ramp rate limits (Hydro only) at each time step |
| Battery energy storage | - Power and energy capacity constraints at each time step - Storage inventory balance constraints with charging/discharging efficiency - Minimum amount of energy in storage in at each time step |
| Electrolyzer | - Capacity constraint with constant availability factor at each time step - Power consumption by electrolyzer associated with H_2_ production in each time step (sector-coupling) - Minimum H_2_ production rate at each time step - Ramping rate limits at each time step |
| H_2_ storage | - Charging power (compression) and energy capacity constraints at each time step - Storage inventory balance constraints with charging/discharging efficiency at each time step - Minimum amount of energy in storage at each time step - Maximum energy storage capacity limit (implemented for some of the evaluated scenarios) |

## S1.2 Key system-level constraints

Aside from the supply-demand balance at each time step for electricity and H_2_ commodities and constraints related to time-matching requirement (TMR) described in the main text, the model includes the following system level constraints.

- **Resource adequacy constraint (Eq. S1)**: this constraint, referred sometimes as the capacity reserve margin, enforces the need to procure “firm” generation capacity in excess of demand ($\delta_{t}$) by the specified amount (i.e. reserve margin) in each hour of the year. Here the firm capacity contribution of each resource, after accounting for a derating factor($\eta)$, is calculated based on: a) the installed capacity ($Y_{g})$ in case of thermal plants ($g\in D$), b) the hourly available generation ($X_{g,t}^{a})$ in case of non-dispatchable resources like renewables and c) the difference between discharging and charging rates ($X_{s,t}^{d}-X_{s,t}^{c})$ in case of energy storage and d) the negative of net consumption ($gen_{r,t}^{ELY}\beta_{r})$ in case of flexible demand (e.g. electrolyzers). Table S5 reports the derating factor assumptions for each resource as well as the enforced capacity reserve margin ($\alpha_{CRM})$.

| $\sum_{g\in D} Y_{g}\eta_{g}+ \sum_{g\in ND} X_{g,t}^{a}\eta_{g}- \sum_{s\in S} \left( X_{s,t}^{d}-X_{s,t}^{c} \right) \eta_{s}-\eta^{ELY}gen_{t}^{ELY}\beta^{ELY} \geq\left( 1+\alpha_{CRM} \right)\delta_{t}$ | (S1) |
| --- | --- |

- **Renewable Portfolio Standard (RPS) requirement (Eq. S2)**: this constraint requires that annual generation from existing and new VRE resources serving the grid and not contracted with the electrolyzer (i.e. so-called ‘grid’ or ‘non-PPA’ resources, $g\in VRE_{g}\backslash TMR_{g}$), must be at least equal to a pre-specified ($\kappa$) of annual electricity demand ($\delta_{t}$). Note that electricity demand does not include electricity consumed for H_2_ production.

| $\sum_{g\in VRE_{g}\backslash TMR_{g}} \sum_{t\in T} gen_{g,t}\geq\kappa\times\sum_{t\in T} \delta_{t}$ | (S2) |
| --- | --- |

## S1.3. Out-of-sample analysis

The out-of-sample analysis takes the capacity decisions obtained from the solution of the stochastic and deterministic model (the *design* models) and tests their performance using VRE availability for other (*out-of-sample model)* VRE scenarios, corresponding to different weather scenarios. The purpose is to assess the robustness of solutions generated by the stochastic and deterministic model, as well as gain insights into possible contract designs for procurement of hourly renewable electricity and need for real-time markets to balance supply and demand for clean energy attribute certificates (EACs). The cost-optimal system design for the power grid and H_2_ production and storage obtained by the *design* model are fixed in the *out-of-sample dispatch* model where the operation of this system is optimized using an *out-of-sample* weather scenario instead of the VRE weather used to generate the design solution.

Without some level of flexibility in the hourly-time matching constraint, the out-of-sample dispatch model may be infeasible. To maintain model feasibility and quantify how much operational flexibility is required, a slack term, $TMR_{slack,t}$, is introduced into the hourly TMR constraint (Eq. 2) for the out-of-sample model runs. This slack term enables the electrolyzer to operate with imperfect matching from contracted resources. Without this slack term, the model would be infeasible whenever contracted VRE resources are unable to meet the TMR constraint. Utilization of $TMR_{slack,t}$ is penalized at $500/MWh in the objective function, which is lower than the cost of not serving the grid ($9000/MWh – See Table S4) but well above the cost of the most expensive electricity generator. This ensures that the slack variable is only used when electricity from the PPA resources is scarce, but that the time matching constraint will not take priority over serving grid demand during grid scarcity events. For the out-of-sample analysis, we focus on the impact of the more stringent hourly-time-matching requirement since this is the more stringent of the two TMR constraints, and because hourly TMR is being considered as part of multiple regulatory and voluntary efforts for characterizing electricity-related emissions of end-users.

## S1.4. Modeling Renewable Portfolio Standards (RPS) and Time-Matching Requirements in the stochastic model

Under the stochastic model, the annual RPS requirement is enforced as a separate constraint for each of the nine weather scenarios. Practically, this means that the resource mix reflected in the model solution must be sufficient to meet the RPS requirement in every weather scenario, and is therefore sized to meet the most challenging weather scenario(s). The consequence is that the annual VRE generation under some scenarios will exceeds the RPS requirement in most scenarios and be binding in a subset of scenarios. Effectively, the shadow price of the RPS requirement constraint in many of the modeled weather scenarios is likely to be zero.

In addition, we do not count generation from PPA resources towards meeting the RPS requirement so as to avoid double counting the clean energy attributes of the resource. In addition, PPA VRE resources are disallowed from contributing to the resource adequacy requirement (see Eq. S1). This ensures that resources designated as PPA are being built purely to serve the TMR requirement constraint associated with the electrolyzer demand, as opposed to being built to serve either the RPS or resource adequacy constraint.

## S1.5 Implementation details

The numerical experiments were run on high-performance computing clusters available to authors at MIT and NYU using Gurobi 11.0.2. For the resulting linear problems, we used the interior point method without crossover, a barrier convergence tolerance of 10^-8^ and 32 threads per model. The maximum memory requirement for any individual model run was 102 GB.

# S2. Additional results


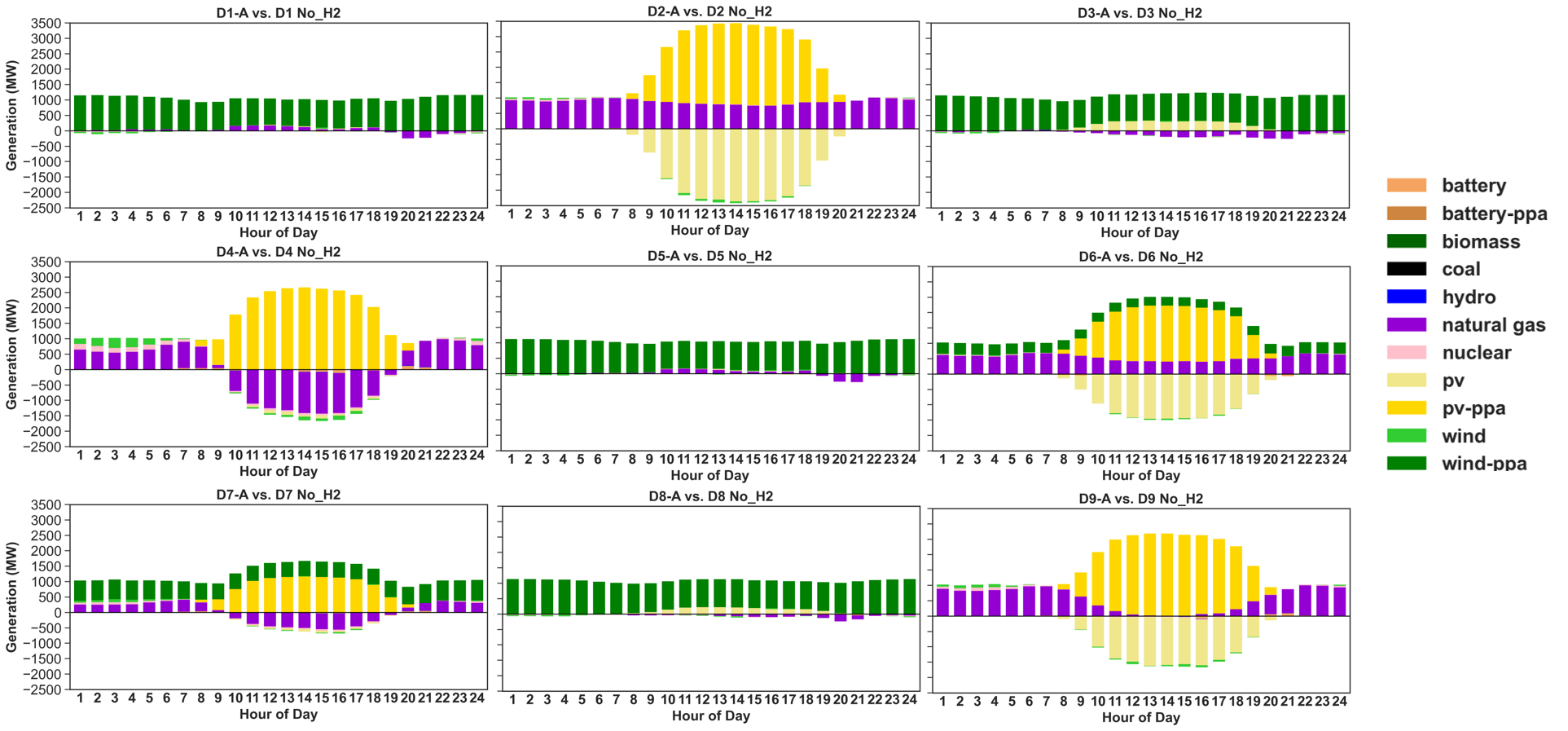


Figure S 1. Difference in average hourly dispatch between counterfactual (with H_2_ demand and time matching requirement –“DX-A”) and baseline grid without H_2_ demand for the deterministic model under different weather scenarios and annual time matching. Each panel is labeled to show results for one weather scenario. DX-A = Deterministic model using weather scenario “X” (1-9) with H_2_ production and annual time matching. DX No_H_2_ = Deterministic using weather scenario “X” (1-9) without H_2_ production and time matching requirement.


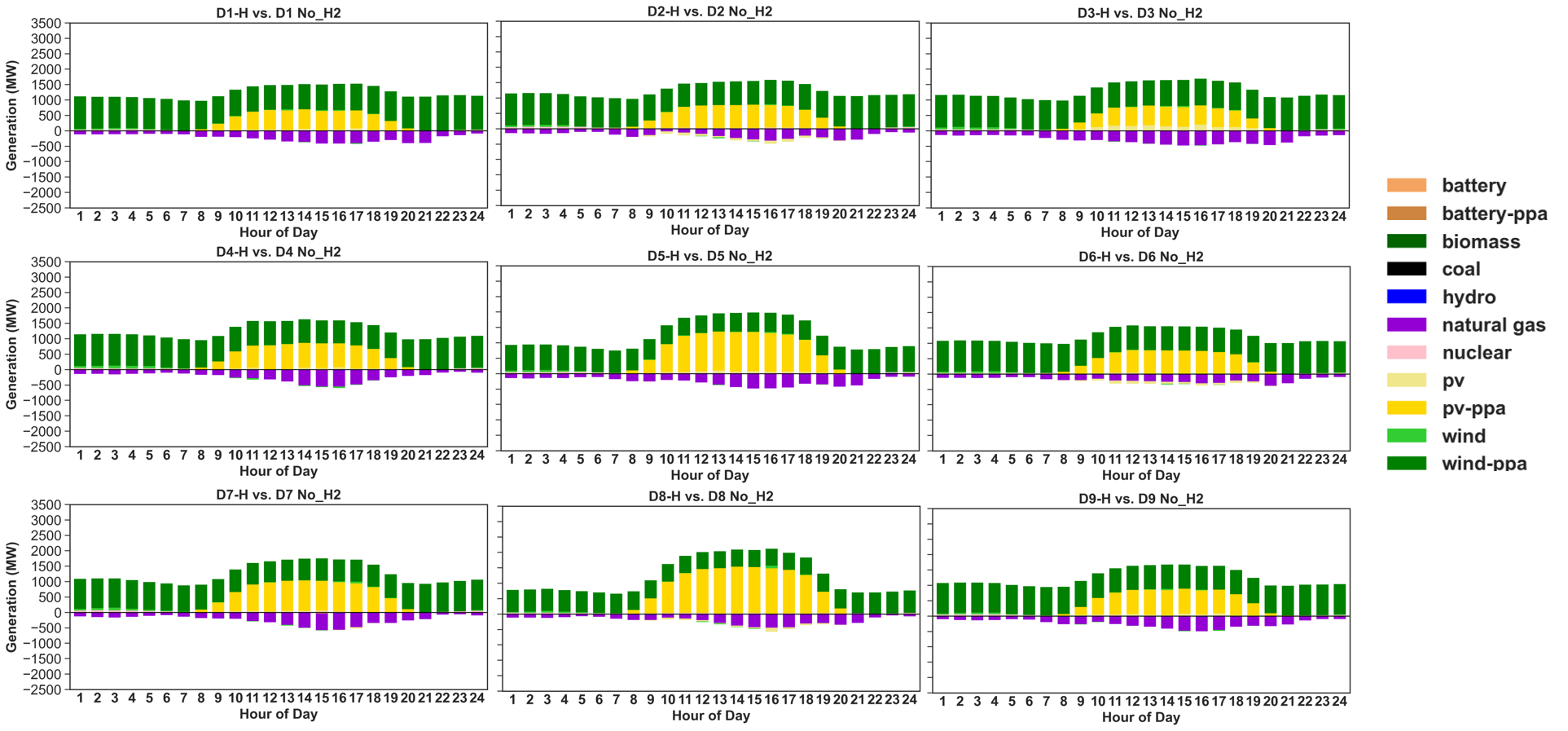


Figure S 2. Difference in average hourly dispatch between counterfactual (with H_2_ demand and time matching requirement –“DX-A”) and baseline grid with no H_2_ demand for the deterministic model under different weather scenarios and hourly time matching. Each panel is labeled to show results for one weather scenario. DX-H = Deterministic model using weather scenario “X” (1-9) with H_2_ production and annual time matching. DX No_H_2_ = Deterministic using weather scenario “X” (1-9) without H_2_ production and time matching requirement.


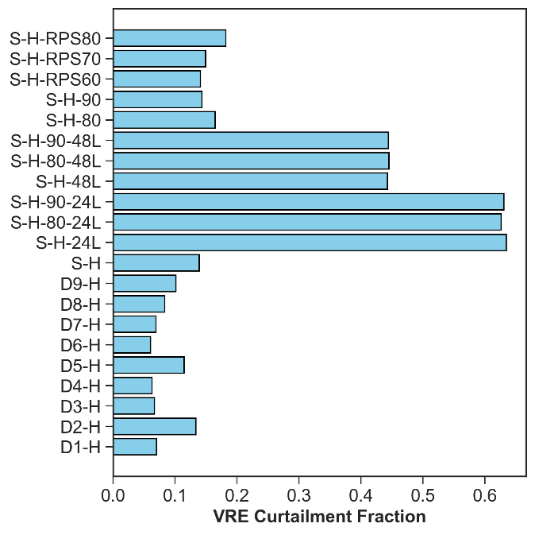


Figure S 3. VRE curtailment fraction for PPA resources across deterministic and stochastic model cases with hourly matching. VRE curtailment defined as the ratio of curtailed energy production from VRE resources to the available energy from VRE resources. DX-H= Deterministic model for weather scenario X (1-9) with hourly matching; S-H= Stochastic model with hourly matching; S-H-X = Stochastic model with hourly matching with X percent (80,90) compliance of the constraint; S-H-X-YL = Stochastic model with hourly matching with X percent compliance (80,90) and limit on H_2_ storage capacity equal to Y hours of H_2_ demand (Y = 24, 48). S-H-YL= Stochastic model with hourly matching with 100 percent compliance and limit on H_2_ storage capacity equal to Y hours of H2 demand (Y = 24, 48). Annual matching scenarios generally have near zero VRE curtailment and hence are not shown.


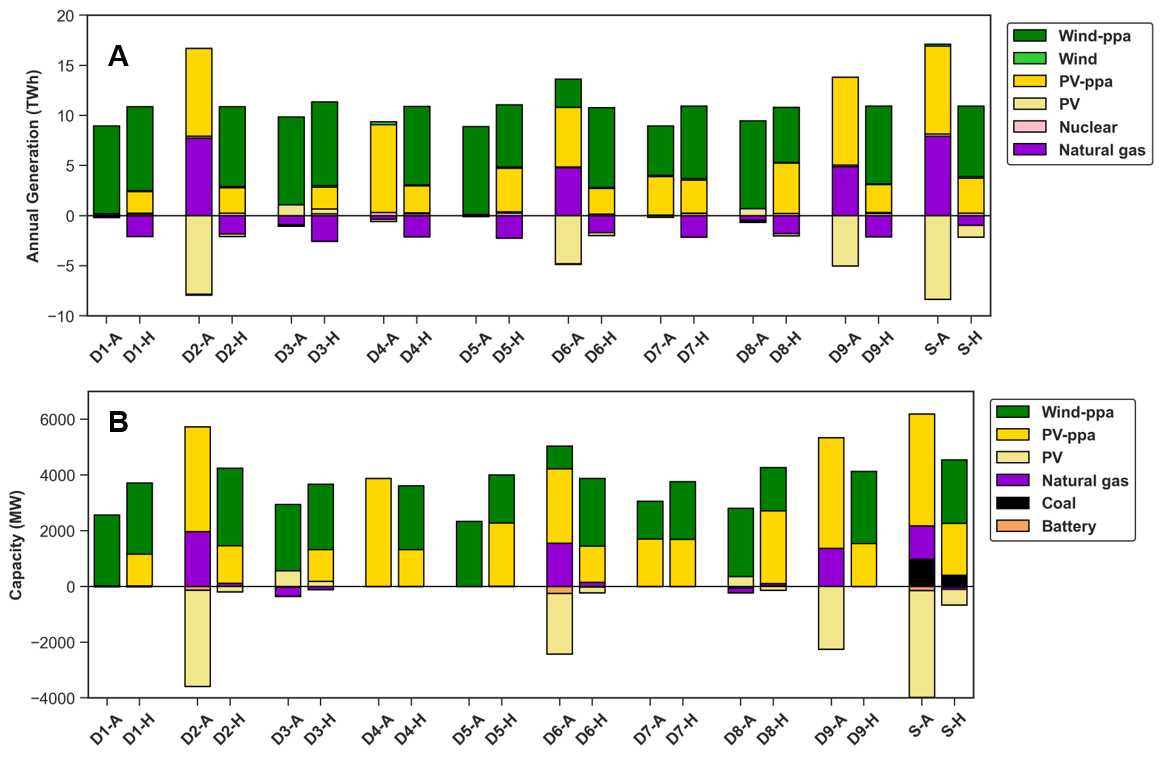


Figure S 4. Difference in annual generation (A) and power generation and storage capacity (B) as a result of electricity-based H_2_ production with annual or hourly TMR under deterministic and stochastic models. Model configuration nomenclature as defined in Table 1 and Figure 1 caption in the main text.


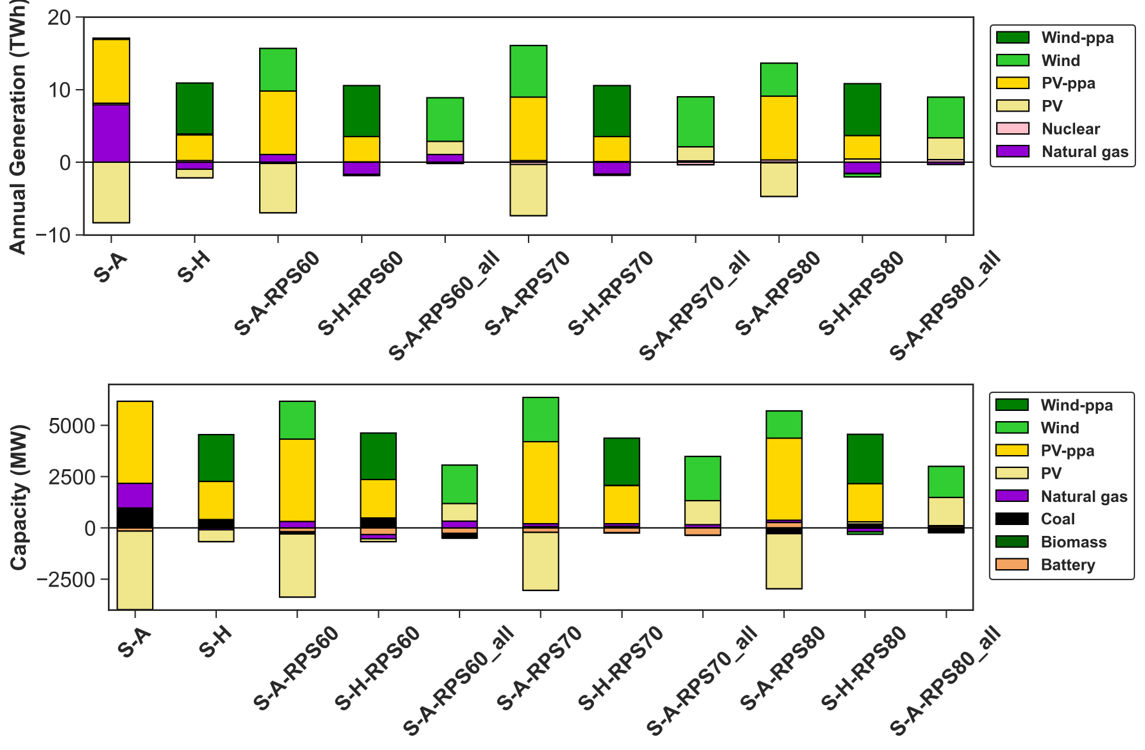


Figure S 5. Difference in annual generation mix (top) and capacity mix (bottom) with and without electricity demand from H_2_ production under different scenarios of time-matching requirements on H2-related electricity matching (no, annual, hourly) and renewable portfolio standard (RPS) requirements (no RPS, 60%, 70%, 80%) on non-H_2_ electricity demands. "RPS 60" indicates 60% minimum annual VRE generation for non-H₂ demand and so on. "RPSX_all" includes electrolyzer consumption in the RPS constraint with no time-matching requirement. S-A/S-H: annual/hourly time-matching and no RPS constraint. S-A-RPSXX/S-H-RPSXX: annual/hourly time-matching for electrolyzer with XX% RPS for non-H₂ demand.


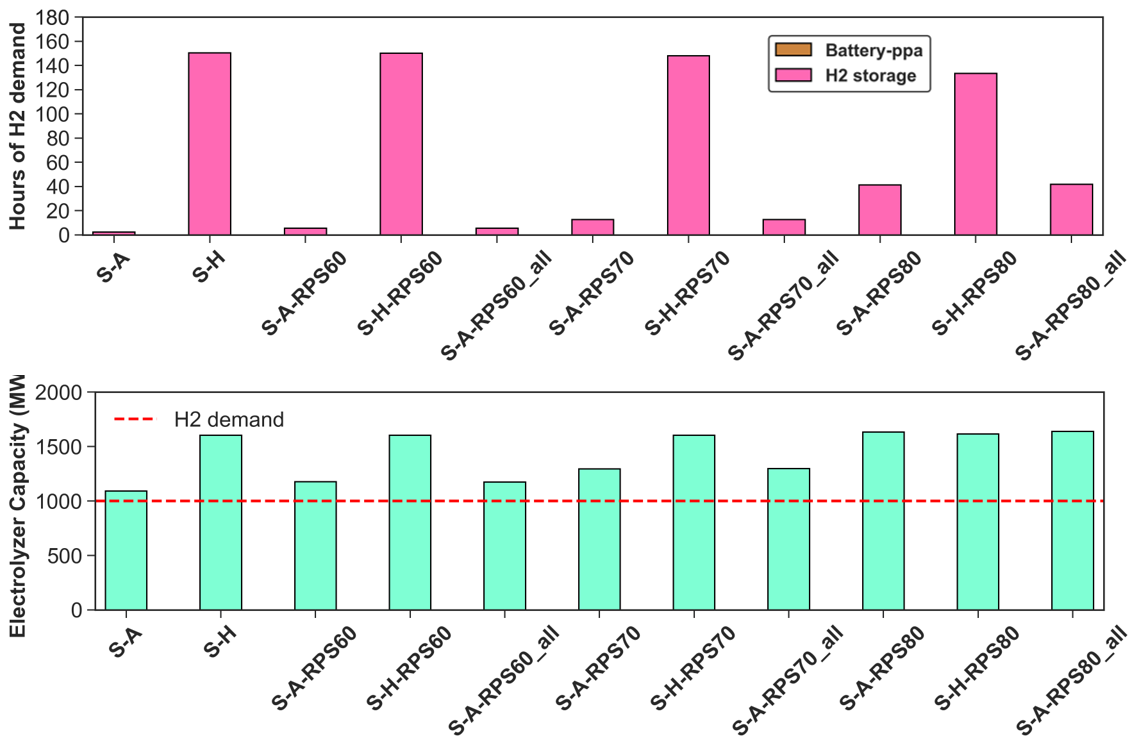


Figure S 6. Installed PPA energy storage capacity (top) and electrolyzer capacity (bottom) to support electrolytic H_2_ production (1 GW) under different scenarios of time-matching requirements (no, annual, hourly) and renewable portfolio standard (RPS) requirements (no RPS, 60%, 70%, 80%) on non-H_2_ electricity demands. "RPS 60" indicates 60% minimum annual VRE generation for non-H₂ demand and so on. "RPSX_all" includes electrolyzer consumption in the RPS constraint with no time-matching requirement. S-A/S-H: annual/hourly time-matching and no RPS constraint. S-A-RPSXX/S-H-RPSXX: annual/hourly time-matching for electrolyzer with XX% RPS for non-H₂ demand. The H_2_ storage capacity is reported in terms of hours of H_2_ demand, which is calculated by dividing the H_2_ storage capacity by the baseload H_2_ demand (18.4 tonnes/hour). No PPA battery energy storage is deployed across the evaluated scenarios. All results shown are for the stochastic model.


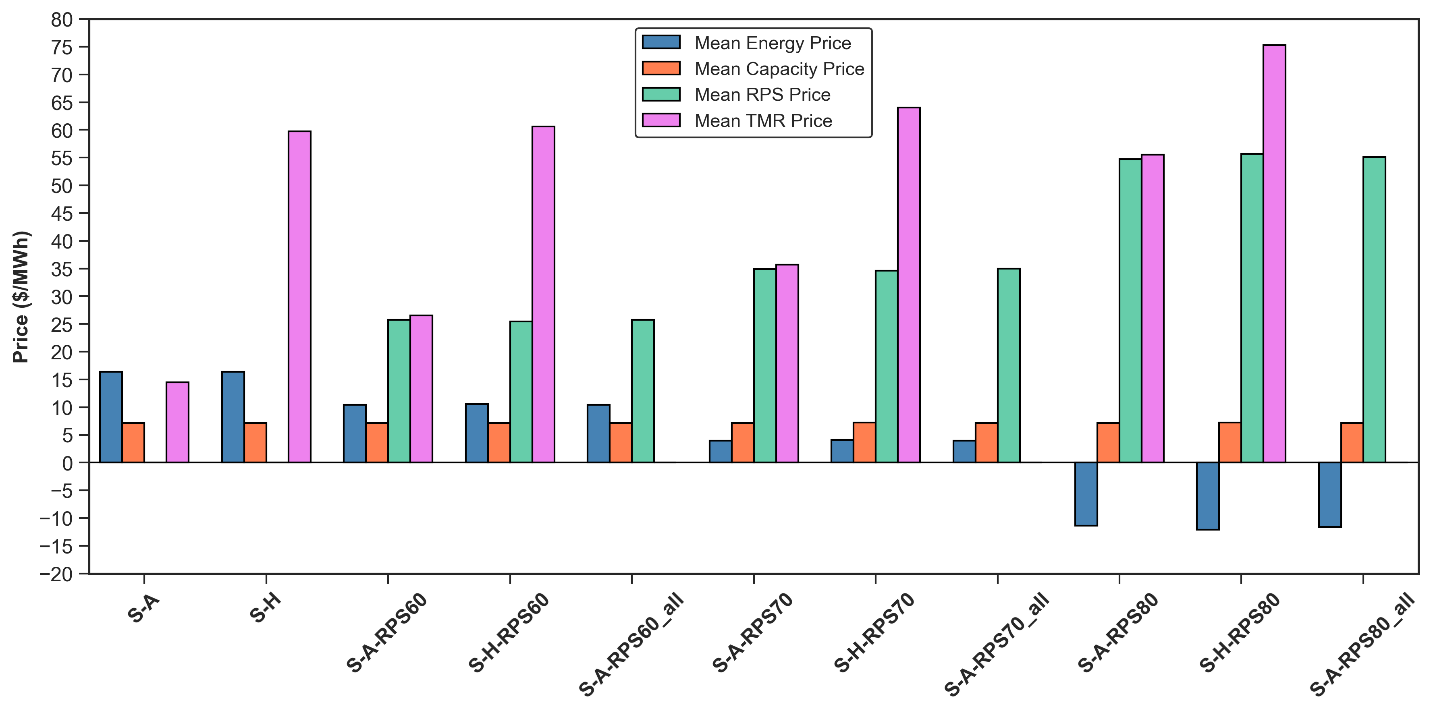


Figure S 7. Average energy price, capacity price, renewable portfolio standard (RPS) price, and time-matching requirement (TMR) price for stochastic model runs with different time-matching requirements (TMR) (no TMR, annual, hourly) as well as RPS constraints (no RPS, 60%, 70%, 80%). See Figure S 5 caption for scenario name explanations. Energy price – shadow price of hourly electricity supply-demand balance constraint, capacity price – shadow price of hourly resource adequacy constraint, RPS price – shadow price of annual RPS constraint, TMR price – shadow price of TMR constraint (annual or hourly depending on the case). RPS = Renewable Portfolio Standard.


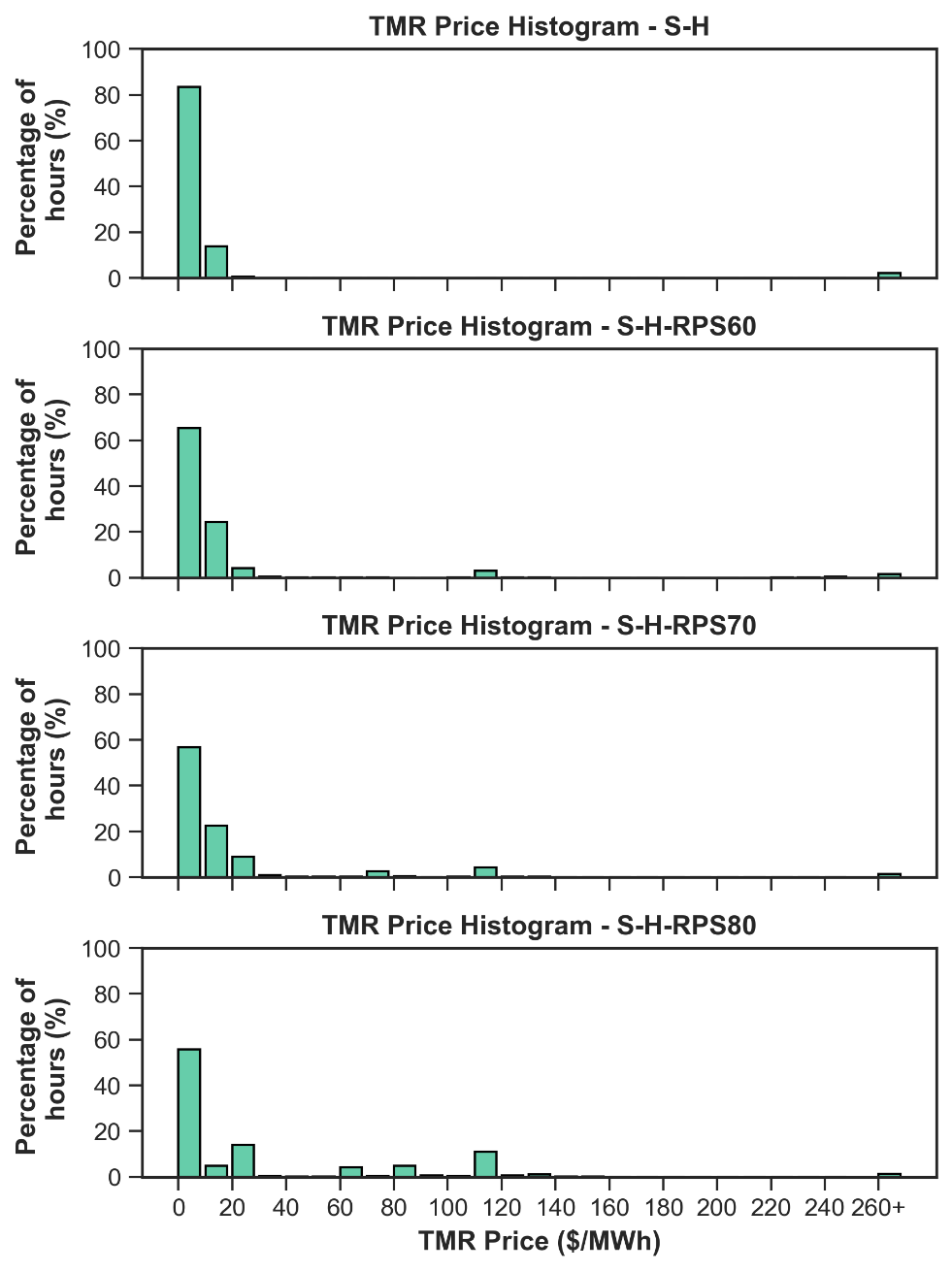


Figure S 8. Distribution of hourly time-matching requirement constraint shadow prices for the stochastic model evaluated under different scenarios of RPS requirement for non-H_2_ (e.g., grid) electricity demand. Prices greater than $260 /MWh are aggregated in the last bin labeled “260+”.

# S3. Model data inputs

## S3.1. VRE Data and Scenario Selection

Both the deterministic and stochastic model use hourly VRE availability profiles from ERCOT as inputs. The deterministic model considers one year of hourly VRE generation (8760 hours), whereas the stochastic model considers nine years of hourly data (9 * 8760 hours) for each technology (wind, solar) by resource type (existing and new).

To construct these profiles, we use the ERCOT’s Hourly Wind and Solar Generation Profiles dataset^4^ which provides hourly solar and wind generation profiles for existing and planned plants from 1980 to 2021. Existing plants are defined as VRE plants that were operational as of 2020. Planned plants are VRE plants that had received approval for or were under construction as of 2020. The ERCOT dataset uses spatially granular historical weather data to estimate hourly generation from both types of resources for all years in the dataset — e.g., a planned resource will still have an hourly generation profile available for 1980 that is based on the technical parameters of that plant and the weather conditions in 1980. To construct the VRE profiles input into our single-region model of ERCOT, we aggregate by existing or planned resources, sum the hourly generation within both groups, and divide the aggregated hourly generation by the total capacity of each group. The result is four time series (existing/planned x wind/solar) of hourly capacity factors for the years 1980-2021(Figure S 9).


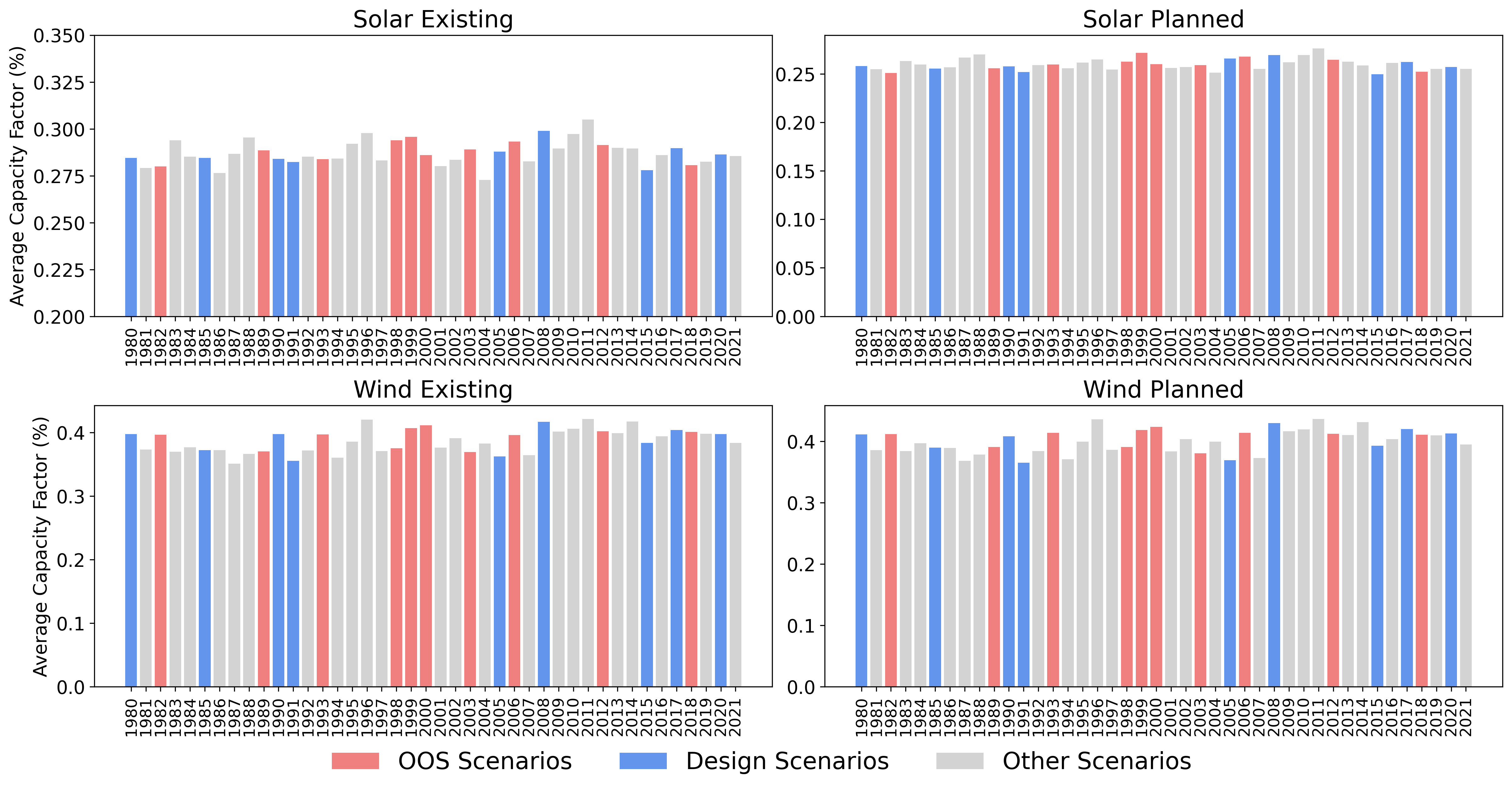


Figure S 9. **Annual averaged capacity factors of solar and wind resources in ERCOT (1980-2021).** Capacity factors are reported for solar (A, B) and wind (C, D) resources that are either existing (A, C) or planned (B, C) as of 2021. Note that the y-axes do not extend to zero, which is done to make it easier to observe variation among years. OoS = Out of sample; Only OoS and Design scenarios are used in the analysis.

To balance the computational resource needs to solve the stochastic model while striving to maximize the extent to which we capture the variability among VRE availability profiles, we perform scenario reduction via k-means clustering to identify nine representative scenarios from the data. As seen in other studies^1,2,5^, wind is favored to meet an hourly time-matching requirement, so we select representative VRE years based on wind. Solar scenarios correspond to the years selected from the k-means clustering for wind. The nine representative VRE scenarios correspond to the years 1980, 1985, 1990, 1991, 2005, 2008, 2015, 2017, and 2020, whose hourly capacity factor distribution for new wind and solar resources is highlighted in Figure S 10A and B, respectively.


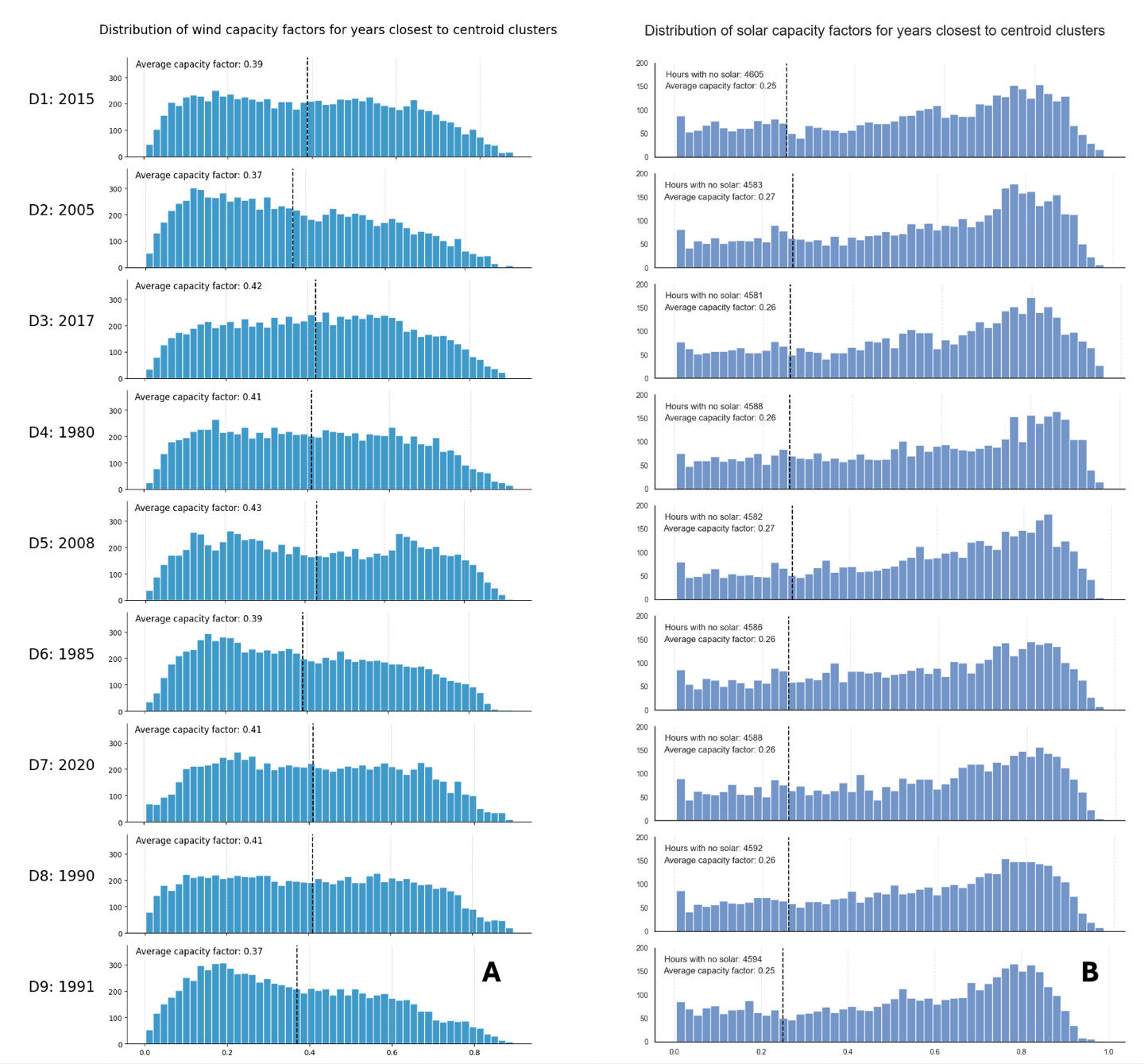


Figure S 10. **Distribution of hourly wind and solar capacity factors for the nine design cases.** Data corresponds to planned wind resources (A) and planned solar resource (B) from the nine VRE years selected via k-means clustering from the 41 ERCOT VRE scenarios. Design scenarios are label “DX” followed by the year of ERCOT’s VRE data that they correspond to. Vertical lines indicate the average capacity factor. To make it easier to see the distribution of hours with non-zero capacity factors for solar, hours with capacity factors of less than 0.005 are not shown in the chart, but the number of such hours is reported as “Hours with no solar” in the top left of each subplot.

The 10 out-of-sample VRE scenarios were selected by randomly sampling from the 31 VRE scenarios that are not used for the design cases. The selected years were 1982, 1989, 1993, 1998, 1999, 2000, 2003, 2006, 2012, and 2018 whose hourly capacity factor distribution for new wind and solar resources is highlighted in Figure S 11A and B, respectively.


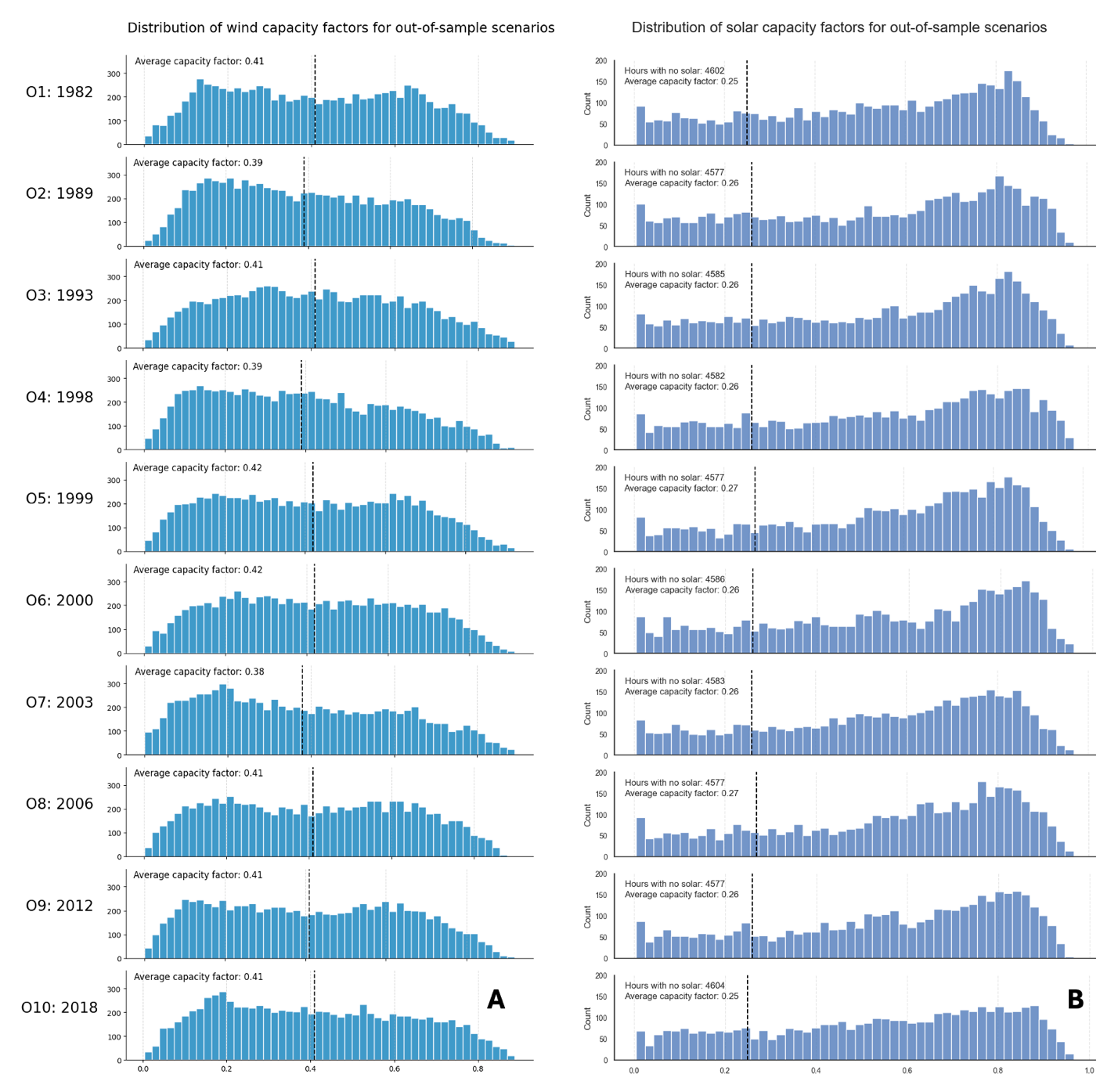


Figure S 11. **Distribution of hourly wind and solar capacity factors for the 10 out-of-sample scenarios.** Data corresponds to planned wind resources (A) and planned solar resource (B) from the 10 VRE years randomly selected from 31 ERCOT VRE scenarios, excluding the years used for the design cases. Out-of-sample scenarios are label “OX” followed by the year of ERCOT’s VRE data that they correspond to. See caption of Figure S9 for further details.

## S3.2. Technology cost and performance assumptions

This section summarizes the major technology cost and performance assumptions. Unless otherwise specified, the stochastic model and deterministic model use the same assumptions as described in our prior work^6^— i.e., technology cost and performance, as well as power system characteristics, such as electricity demand, existing generators, value of lost load.

All costs are reported on a 2021 USD. Table S2 summarizes the cost assumptions for VRE, natural gas and Li-ion battery storage resources. Battery storage has a self-discharge rate of 0.002% per hour^7^. The model can independently vary the installed energy capacity and power capacity for Li-ion storage so long as the ratio of energy capacity to power capacity (i.e., duration) is between 0.15-12 hours. Table S3 summarizes cost assumptions for electrolyzers and H_2_ storage. Fuel prices correspond to 2019 data adjusted to 2021 USD, due to COVID-related disruptions to fuel prices that occurred in 2021.

Table S2. Electricity generation and storage technology cost and operational parameters. Annualized investment costs are calculated with a discount rate of 4%. Cost assumptions are adapted from the NREL Annual Technology Baseline (ATB) 2022 edition^8^. The annualized capex values reported in the table include a 30% discount to approximate the investment tax credit for wind, solar and storage consistent with policy as per the U.S. inflation reduction act of 2022. Li-ion battery storage is subject to a variable operation and storage cost to discourage simultaneous charging and discharging, which must be explicitly discouraged a linear programming capacity expansion model. NGCT = Natural Gas combustion turbine. NGCC = Natural gas combined cycle.

| **Technology** | | **Solar PV** | **Onshore wind** | **Li-ion battery storage** | **NGCT** | **NGCC** |
| --- | --- | --- | --- | --- | --- | --- |
| Lifetime (years) | | 30 | 30 | 15 | 30 | 30 |
| Investment cost – power ($/MW) | | 1,176,000 | 1,428,000 | 255,150 | 950,249 | 1,080,449 |
| Annualized CAPEX  – Power ($/MW/year) | | 47,606 | 57,807 | 16,064 |  |  |
| Investment cost – energy ($/MWh) | | - | - | 296,100 |  |  |
| Annualized CAPEX – Energy ($/MWh/year) | | - | - | 18,642 | - |  |
| Fixed operation and maintenance cost | Power ($/MW/year) | 21,000 | 44,100 | 6,379 | 11,849 | 13,513 |
|  | Energy ($/MWh/year) | - | - | 7,403 |  |  |
| Variable operating cost ($/MWh) | | 0.00 | 0.00 | 1.00 | 5.00 | 2.00 |
| Heat rate (MMBtu/MWh) | | - | - | - | 9.71 | 6.36 |
| Start-up fuel use (MMBtu per start) | | - | - | - | 815.5 | 1454.0 |
| Start cost ($/start) | | - | - | - | 27,028 | 64,703 |

Table S3. Hydrogen electrolyzer and storage cost and operation parameters. Annualized investment costs are calculated with a discount rate of 4%. Electrolyzer assumptions reflect 2022 assumptions from the NREL H2A report^9^ , and hydrogen storage assumptions are from Papadias and Ahluwalia (2021). Costs reported in $/MWh are calculated by multiplying $/tonne H2 by the lower heating value of hydrogen. Water costs are assumed to be negligible and therefore are not considered. t = tonne; y = year.

| **Technology** | **Lifetime** | **Investment cost** | | **Annualized investment cost** | | **Fixed operation and maintenance (FOM) cost -H_2_ production rate ($/MWH_2_/year)** | **Electrical power use (MWh/t H_2_)** |
| --- | --- | --- | --- | --- | --- | --- | --- |
|  |  | **H2 production rate ($/MWH_2_)** | **Energy ($/t H_2_)** | **H_2_ Production rate ($/MWH_2_/y)** | **Energy ($/t H_2_/y)** |  |  |
| Electrolyzer | 20 | 1,937,791 | - | 142,586 | - | 28,604 | 54.3 |
| H_2_ storage (tank) | 30 | - | 587,000 | - | 33,929 | - | - |
| H_2_ storage compressor | 15 | 2,451,496 | - | 220,490 | - | - | 0.71 |

Table S4. Global parameters. Fuel prices are from the EIA 2022 Annual Energy Outlook 2022. Natural gas and coal modeled with CO_2_ emissions factors of 0.05306 tCO_2_/MMBtu and 0.09552 tCO_2_/MMBtu, respectively. The capacity reserve margin is selected based on minimum target used by ERCOT for planning purposes. Slack penalties are reported for all constraints where a slack variable is applied. The high penalty for unmet H_2_ and power demand was selected to avoid any load shedding instances. The unserved grid load penalty is based on the value of lost load previously used in ERCOT.

|  | **Value** | **Applicable Cases** |
| --- | --- | --- |
| **Discount Rate used for annualization of capital costs** | 4% | All |
| **Capacity Reserve Margin (used in Eq. S1)** | 13.75% | All |
| **Fuel Prices ($/MMBtu)** |  |  |
| Natural Gas | 2.03 | All |
| Coal | 2.47 | All |
| Uranium (nuclear) | 0.70 | All |
| **Penalties for slack variables in various constraints** |  |  |
| Unserved H_2_ demand ($/tonne H_2_) | 5e+07 | All |
| Unserved power demand ($/MWh) | 9,000 | All |
| Unmet RPS requirement ($/MWh) | 1,000 | RPS cases |
| Unmet Time-matching requirement ($/MWh) | 500 | Out-of-sample cases with a hourly time-matching requirement for hydrogen |

Table S5. Capacity reserve margin derating factors by resource type. Source: prior modeling study^10^. Refer to Eq. S1 and Github repository^3^ for implementation of constraint.

|  | Capacity Reserve Derate Factor (see Eq. S1) |
| --- | --- |
| Coal | 0.93 |
| Natural gas combined cycle | 0.93 |
| Natural gas combustion turbine | 0.93 |
| Nuclear | 0.93 |
| NG steam turbine | 0.93 |
| Biomass | 0.90 |
| Hydro | 0.80 |
| Solar | 0.80 |
| Wind (onshore) | 0.80 |
| Diurnal battery storage | 0.80 |

## S3.3 Existing generator fleet

To increase the number of weather scenarios considered in the stochastic model while maintaining computational tractability with off-the-shelf LP solvers (e.g., Gurobi), we reduced the resolution of the characterization of the existing power generation fleet, sourced from the PowerGenome data base^11^. Specifically, we combined all coal and natural gas steam turbines that either operated at <5% capacity factor in the baseline run (i.e. without any H_2_ demand) from the previous analysis or had heat rates greater than 15 MMBTU/MWh into one cluster. This reduced the total number of generators (including existing and candidate new) from 64 to 49, which enabled more weather scenarios to be considered in the stochastic model.

Table S6. Capacity and maximum hourly availability of generators. Dispatchable fossil, nuclear, and biomass generators are assumed to experience outages, maintenance, etc. resulting in less than 100% availability at all hours. Hydro, solar, and wind availability are assumed to be subject to weather conditions and therefore their maximum hourly variability is time-dependent and separately specified.

|  | Capacity (GW) | Maximum Hourly Availability Factor (%) |
| --- | --- | --- |
| Coal | 7.0 | 90 |
| Natural gas combined cycle | 35.13 | 90 |
| Natural gas combustion turbine | 6.83 | 90 |
| Nuclear | 4.98 | 95 |
| Natural gas steam turbine | 10.23 | 90 |
| Biomass | 0.07 | 90 |
| Hydro | 0.5 | N/A |
| Solar | 9.14 | N/A |
| Wind (onshore) | 34.06 | N/A |

# References

(1) Zeyen, E.; Riepin, I.; Brown, T. Temporal Regulation of Renewable Supply for Electrolytic Hydrogen. *Environ. Res. Lett.* **2024**, *19* (2), 024034. https://doi.org/10.1088/1748-9326/ad2239.

(2) Xu, Q.; Ricks, W.; Manocha, A.; Patankar, N.; Jenkins, J. D. System-Level Impacts of Voluntary Carbon-Free Electricity Procurement Strategies. *Joule* **2024**, *8* (2), 374–400. https://doi.org/10.1016/j.joule.2023.12.007.

(3) DOLPHYN Model, 2023. https://github.com/macroenergy/DOLPHYN (accessed 2023-02-13).

(4) *Resource Adequacy 2022*. https://www.ercot.com/gridinfo/resource/2022 (accessed 2024-05-07).

(5) Ricks, W.; Xu, Q.; Jenkins, J. D. Minimizing Emissions from Grid-Based Hydrogen Production in the United States. *Environ. Res. Lett.* **2023**, *18* (1), 014025. https://doi.org/10.1088/1748-9326/acacb5.

(6) Giovanniello, M. A.; Cybulsky, A. N.; Schittekatte, T.; Mallapragada, D. S. The Influence of Additionality and Time-Matching Requirements on the Emissions from Grid-Connected Hydrogen Production. *Nat. Energy* **2024**, *9* (2), 197–207. https://doi.org/10.1038/s41560-023-01435-0.

(7) *The Future of Energy Storage*. Main. https://energy.mit.edu/publication/the-future-of-energy-storage/ (accessed 2022-07-27).

(8) National Renewable Energy Laboratory (NREL). *Annual Technology Baseline*. https://atb.nrel.gov/electricity/2022/data (accessed 2023-05-14).

(9) James, Brian; Colella, Whitney; Moton, Jennie; Saur, G; Ramsden, T. *PEM Electrolysis H2A Production Case Study Documentation*; U.S. Department of Energy: Washington, DC, 2013. https://www.hydrogen.energy.gov/pdfs/h2a_pem_electrolysis_case_study_documentation.pdf.

(10) Law, J. W.; Mignone, B. K.; Mallapragada, D. S. Role of Technology Flexibility and Grid Coupling on Hydrogen Deployment in Net-Zero Energy Systems. *Environ. Sci. Technol.* **2025**, *59* (10), 4974–4988. https://doi.org/10.1021/acs.est.4c12166.

(11) *PowerGenome*. https://github.com/PowerGenome (accessed 2024-08-31).
